# Probing Active Sites on Pd/Pt Alloy Nanoparticles by CO Adsorption

**DOI:** 10.1021/acsnano.4c08291

**Published:** 2024-11-02

**Authors:** Daniel Silvan Dolling, Jiachen Chen, Jan-Christian Schober, Marcus Creutzburg, Arno Jeromin, Vedran Vonk, Dmitry I. Sharapa, Thomas F. Keller, Philipp N. Plessow, Heshmat Noei, Andreas Stierle

**Affiliations:** † Centre for X-ray and Nano Science CXNS, Deutsches Elektronen-Synchrotron DESY, 22607 Hamburg, Germany; ‡ Fachbereich Physik, Universität Hamburg, 20355 Hamburg, Germany; § Institute of Catalysis Research and Technology (IKFT), Karlsruhe Institute of Technology (KIT), 76344 Eggenstein-Leopoldshafen, Germany

**Keywords:** FT-IRRAS, nanoparticle, Al_2_O_3_, PdPt, alloy, CO adsorption, DFT calculations

## Abstract

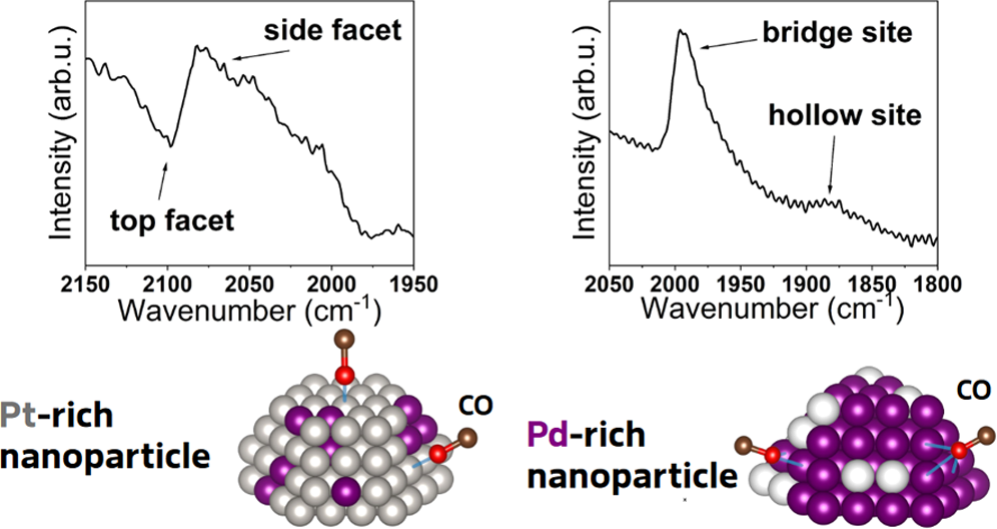

We studied the adsorption of CO on Pd/Pt nanoparticles (NPs) with varying compositions using polarization-dependent Fourier transform infrared reflection absorption spectroscopy (FT-IRRAS) and theoretical calculations (DFT). We prepared PtPd alloy NPs via physical vapor codeposition on α-Al_2_O_3_(0001) supports. Our morphological and structural characterization by scanning electron microscopy and grazing incidence X-ray diffraction revealed well-defined, epitaxial NPs. We used CO as a probe molecule to identify the particles’ surface active sites. Polarization-dependent FT-IRRAS enabled us to distinguish CO adsorption on top and side facets of the NPs. The role of the Pd/Pt alloy ratio on CO adsorption was investigated by comparing the experimental CO stretching band frequency for different alloy arrangements to the results for pure Pd and Pt NPs. Moreover, we studied the influence of hydrogen adsorption on the NP surface composition. We determined the dependence of the IR bands on the local atomic arrangement via DFT calculations, revealing that both bulk alloy composition and neighboring atoms influence the wavenumber of the bands.

Pd/Pt alloy nanoparticles (NPs) are most prominently used for emission control in applications involving CO and methane oxidation, as well as catalysts in fuel cells.^1,2^ The use of an alloy increases the stability of the NPs against aging: Pure Pt NPs sinter and decrease in their catalytic efficiency in the catalysis process, but the addition of Pd slows this process down, making the catalysts last longer^3−6^ and possibly increases the catalytic activity.^7^ In emission control, Pd/Pt NPs are often supported by Al_2_O_3_ due to its good thermal and mechanical stability as well as the high surface area that is achieved when a porous Al_2_O_3_ structure is used.^5,8^ The catalytic activity is controlled by the active surface sites of the NPs.^9^ To study the active sites, model systems with good control over all structural parameters are needed. Here, NPs are grown onto α-Al_2_O_3_ single-crystal substrates and are probed by CO adsorption. Similar model catalysts were employed in the past to study the pressure and coverage dependence^10^ of CO adsorption on Pd NPs. The adsorption of CO molecules on metal surfaces weakens the bond between carbon and oxygen, resulting in a shift in the vibrational frequency that can be probed by Fourier transform infrared reflection absorption spectroscopy (FT-IRRAS).^11,12^ The frequency shift reflects the chemical environments at the catalyst surface, as well as the adsorption site. CO can adsorb on metal surfaces in different configurations, most of them fall into one of three categories: on-top, bridge or hollow adsorption sites. It is well established that at room temperature (RT) Pt surfaces favor on-top adsorption of CO,^13,14^ whereas Pd surfaces mostly give rise to bridge- and hollow-site adsorption.^15,16^ At lower temperatures (and higher coverages) on-top adsorption of CO on Pd and bridge site adsorption on Pt is possible. An overview of literature values of CO vibrational frequencies on different adsorption sites on Pd and Pt (single crystals and NPs^34−41^) can be seen in Figure 1: The purple area marks the on-top adsorption, yielding a large wavenumber region for Pt. The region of bridge site adsorption is marked green and features distinctively different wavenumbers for Pd and Pt. The hollow site adsorption on Pd is marked blue; no hollow site adsorption on Pt is reported. Furthermore, the adsorption configuration may depend on the coverage.^10^ As a trend, the wavenumber to excite a vibrational state of the CO adsorbed on a bridge site is larger than on a hollow site, and the wavenumber corresponding to adsorption on-top is larger than the wavenumber corresponding to adsorption on bridge sites.^13,17−19^ In many cases, the excitation wavenumber for CO increases with increasing coverage due to dipole–dipole coupling in between adsorbed CO molecules and due to changes in the chemical bond in the presence of several molecules.^20−23^ For NPs, the molecular vibration of CO is influenced by the coordination number of the adsorption site, specifically considering adsorption on edges compared to adsorption on facets.^18,20,24^ From theoretical and experimental investigations it is known that CO adsorption on Pt steps and edges results in lower vibration wavenumbers compared to adsorption on facets.^25,26^ On the contrary, for the adsorption of CO on Pd, the wavenumber of the molecular vibration of CO increases when the molecule adsorbs on a low coordinated site.^10,16^ Moreover, in the case of NPs, CO adsorption on the support may also take place.^27^ In the case of alloy NPs, additional phenomena take place, which influence the CO adsorption behavior: density function theory (DFT) calculations predict that the Pd/Pt alloy surface termination depends on the bulk alloy composition and the surrounding atmosphere.^28^ Under ultrahigh vacuum (UHV), for Pd-rich alloys the formation of a Pd shell is energetically favorable, whereas it is favorable to form a Pt shell if the alloy is Pt-rich.^28^ If an oxygen adlayer is present, Pd segregation is energetically advantageous due to its high affinity toward oxygen adsorption. Experimental investigations regarding the surface segregation of Pd/Pt alloy NPs report a preference for Pd segregation in UHV, H_2_, and oxygen.^29−31^ In this work, Pd/Pt alloy NPs grown on α-Al_2_O_3_ are used as model catalysts. The interaction of the CO molecules with different adsorption sites was investigated using polarization-dependent FT-IRRAS, a powerful tool to monitor the interaction of CO with the active sites of Pt/Pd and to gain information about the surface composition of the alloy. Moreover, the effect of different H_2_ and O_2_ pretreatments on adsorption sites and segregation to the alloy surface was studied. The structure and morphology of the NPs were investigated by X-ray diffraction (XRD) and scanning electron microscopy (SEM). The experimental results are combined with DFT calculations, predicting the active surface sites and the CO vibration frequencies as a function of the NP composition.

**Figure 1:**
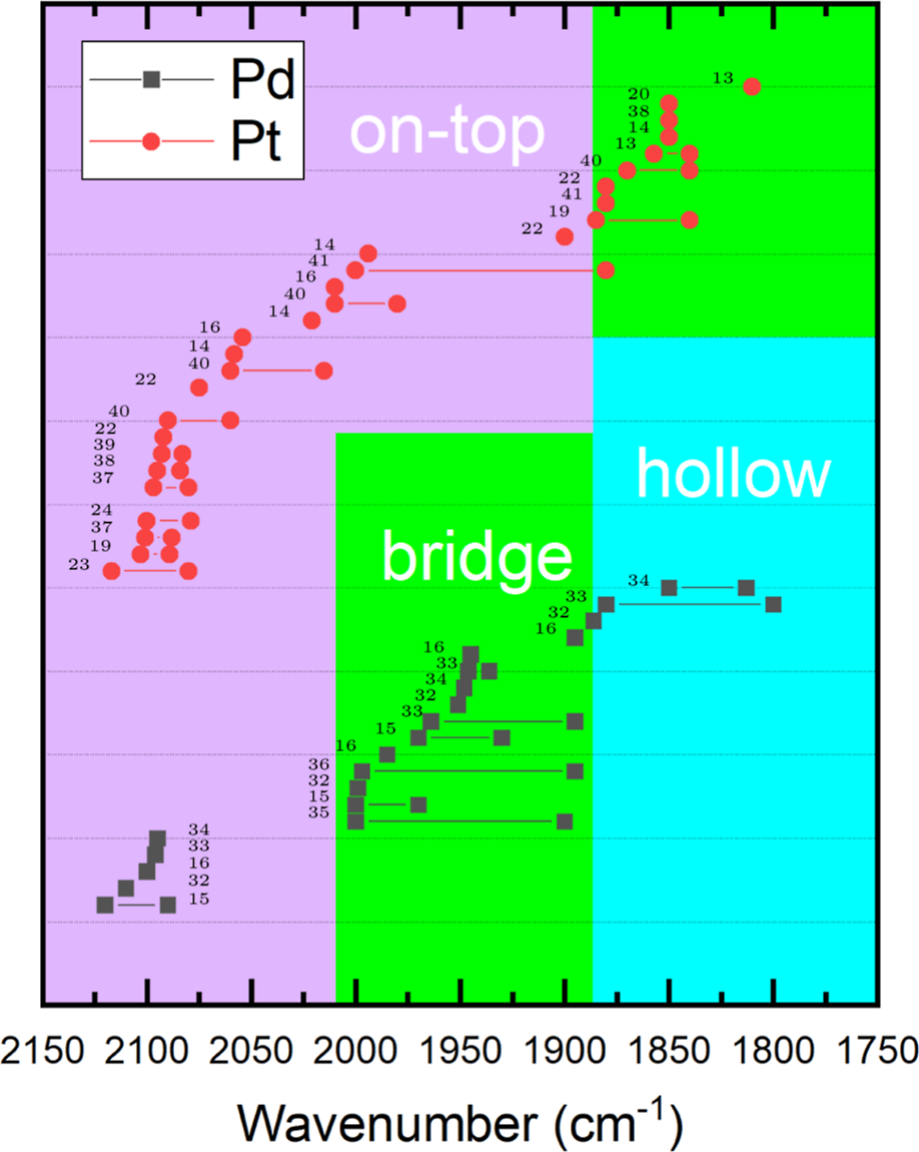
Wavenumbers for specific adsorption sites for CO on single crystals and Pd/Pt NPs. The purple background marks the region of on-top adsorption sites, the green background marks the region of bridge site adsorption and the blue background indicates the region of hollow site adsorption. The data points were taken from literature and can be related to the corresponding publication by their indicated citation number.

## Results and Discussion

### Growth and Structural Characterization of the NPs

The NPs were grown in ultrahigh-vacuum (UHV) using simultaneous Pt and Pd deposition by e-beam evaporation on α-Al_2_O_3_(0001) single crystals at a substrate temperature of 400–450 °C (see Materials and Methods section for details). NPs with defined compositions were prepared by adjusting the calibrated fluxes of Pd and Pt individually. The NPs were characterized using grazing incidence XRD to reveal their orientation, XRR to determine the particle height and coverage and SEM to investigate the lateral NP size. The results from these measurements are combined in Table 1 and all data can be found in the Supporting Information (Figures S6–S11). The particles are about 20 nm in diameter and have heights between 3 and 9 nm. The coverage is roughly 70% with outlayers going up to almost full coverage. It is expected that Pd and Pt are fully mixed in the NPs.^42^ XRD in-plane rocking scans were used to detect signals corresponding to (111), (220) and (200) planes. For the alloys, Vegard’s law was used to calculate the Bragg angles from the lattice constants (*a*_Pd_ = 3.887 Å,^43^*a*_Pt_ = 3.920 Å^44^). All samples feature 60° spaced peaks in the (220) in-plane rocking scans [characteristic for the hexagonal fcc(111) surface lattice] and do not exhibit other peaks. Hence, we conclude that the particles are all (111) oriented (out of plane), see Figures S6–S11. It is known that for both Pt and Pd this results in Pt(220) being parallel to Al_2_O_3_(033̅0).^45,46^ As both Pd and Pt have fcc-type unit cells, this orientation results in NPs exhibiting mainly {111} and {100} type side facets, if considering only low-index facets.^47,48^

**Table 1:** Calculated Values for the NPs Height, Diameter, and Coverage, as Well as the Amount of Nominally Deposited Material and the Growth Temperature^a^

sample	nominally deposited material [nm]	height [nm]	mean diameter [nm]	coverage [ %]	aspect ratio height/diameter	growth temperature [°C]
Pt	1.38 ± 0.14	3.85 ± 0.39	19 ± 2	48	0.20	450
Pd_3_Pt_4_	2.71 ± 0.27	3.61 ± 0.36	30 ± 3	77	0.12	400
PdPt	3.36 ± 0.34	8.81 ± 0.88	32 ± 4	46	0.28	400
Pd_2_Pt	3.28 ± 0.33	4.06 ± 0.40	not defined	84	not defined	400
Pd_3_Pt	2.76 ± 0.28	7.28 ± 0.73	23 ± 3	44	0.48	450
Pd	4.44 ± 0.44	8.38 ± 0.84	21 ± 2	57	0.57	450

a Nominally deposited material, height and coverage were determined via XRR, and the diameter was obtained from the SEM images.

### FT-IRRAS Results

**Figure 2:**
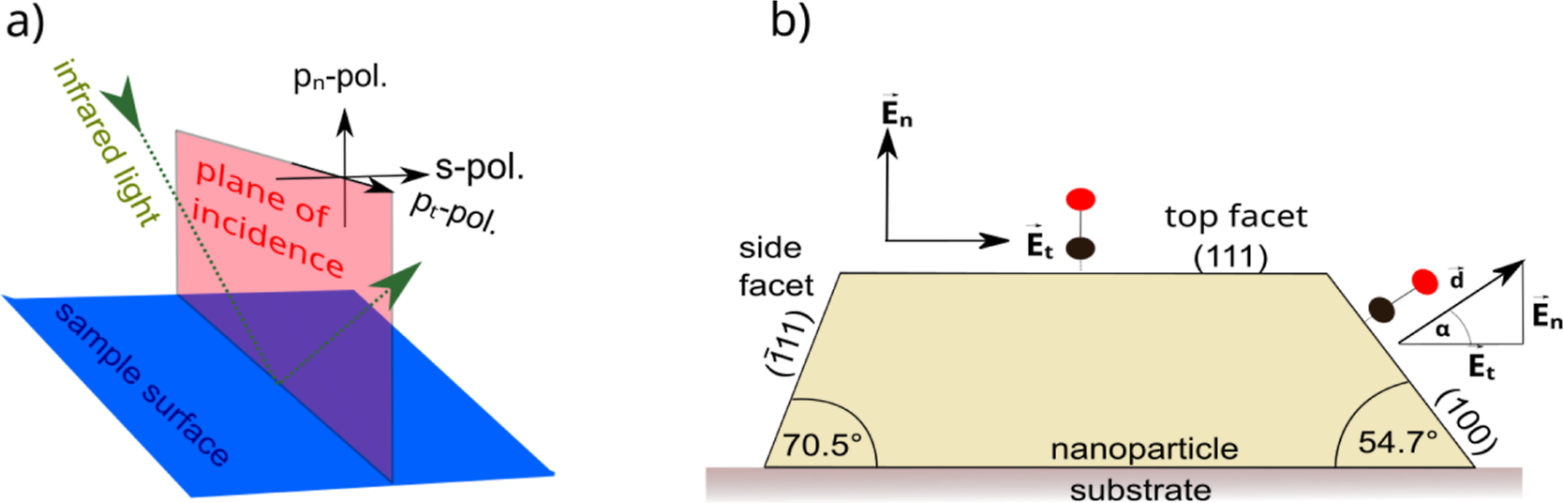
(a) The incoming light can be decomposed into its p-polarized and its s-polarized part. (b) The adsorption site on the NP determines which parts of the light can excite the CO molecular vibration.

Infrared spectroscopy experiments can be performed in s- or p-polarization to excite only specifically oriented adsorbed species. A graphical representation of these polarizations is given in Figure 2. The light polarized parallel to the plane of incidence, p-polarized-light, can be decomposed further into its part parallel to the surface (p_*t*_) and its part perpendicular to the surface (p_*n*_). The light polarized perpendicular to the plane of incidence is the s-polarized component of the light. We consider the Al_2_O_3_ substrate as a reference surface since the wavelength of the IR light is much larger than the NP size. The intensity of the FT-IRRAS signals is proportional to , with α being the angle between the dynamical dipole moment of CO d⃗, and the electric field E⃗. Because the dipole selection rule for metal surfaces allows only detectable dynamic dipole moments perpendicular to the respective metal surface, only dipole moments perpendicular to the NP facets can be probed. Possible configurations can be seen in Table 2, it is always assumed that the NPs have their top (111) facet parallel to the substrate surface. The sign of an FT-IRRAS absorbance signal depends on the substrate and the light polarization.^49^ However, p_*n*_- and s-polarization signals are expected to have the opposite sign compared to p_*t*_-polarization.^50^ For the system used here (metal NPs/Al_2_O_3_), it is observed to be positive for p_*t*_-polarization and negative for p_*n*_- and s-polarization. In this work, the main interest regarding light polarization is the possibility to distinguish signals from NP side facets, with a dipole moment partially parallel to the substrate surface (p_*t*_- and s-polarization), from signals from the top facets with a dipole moment normal to the substrate (p_*n*_-polarization). Thus, the IR bands are labeled as p_*n*_-type, p_*t*_-type or s-type. A similar approach for the identification of CO adsorption on NPs by sum-frequency-generation (SFG) spectroscopy was presented recently.^51^

**Table 2:** Dependence of the FT-IRRAS Signal on the Light Polarization and the Facet the CO Molecule is Adsorbed on, Assuming Perpendicular Orientation of the Molecule to the Surface^a^

facet	E-field	α	sign	cos α
(111)	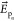	0	-	1
(111)	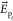	90	+	0
(111)	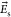	90	-	0
(111̅)	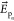	71	-	0.33
(111̅)	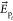	19	+	0.95
(111̅)	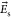	19	-	0.95
(100)	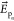	54	-	0.54
(100)	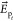	36	+	0.81
(100)	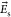	36	-	0.81

a The NP is always assumed to be (111) oriented.

**Figure 3:**
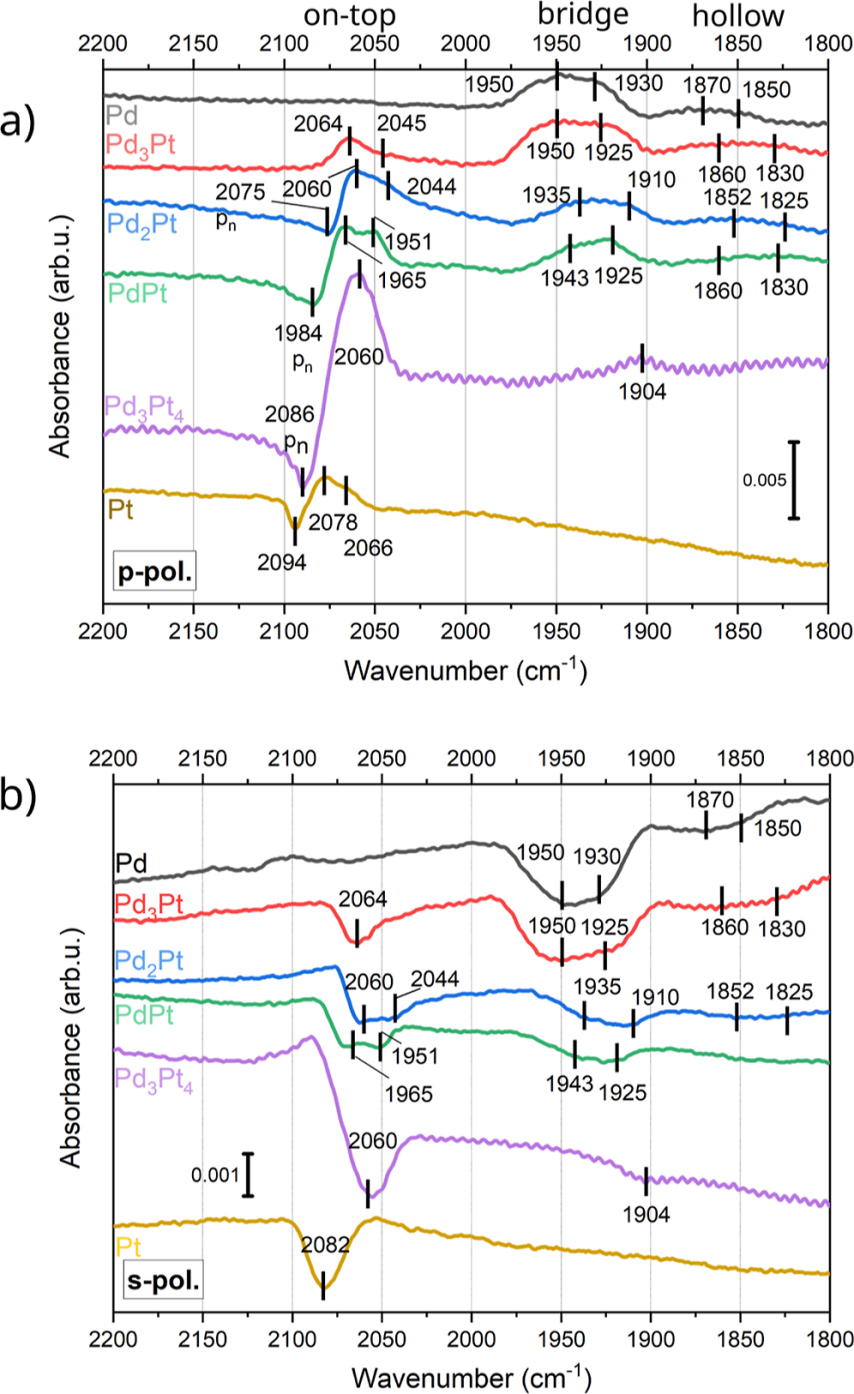
CO adsorption (5 L, dosed at 10^–8^ mbar partial pressure) at RT for the different alloy compositions, with (a) p-polarized and (b) s-polarized light. In (a), all signals that are originating from the p_*n*_ part of the light are marked.

First, we will discuss the room temperature (RT) saturation CO coverage spectra and their dependence on the alloy composition, starting with the pure metal NPs. The spectra are plotted in Figure 3, the exposure-dependent spectra can be found in the Supporting Information (Figures S13–S24), the band assignment for all samples is summarized in Table 3. On pure Pt NPs, three distinct bands can be identified in the IR spectra, all assigned to CO bound to the on-top sites of platinum by comparison with known band positions for Pt from literature, see Figure 1. At 2066 and 2078 cm^–1^ in p-polarization, two positive peaks are found. In s-polarization a corresponding negative band is found at 2082 cm^–1^. As described above, this reveals them as belonging to CO adsorbed on side facets which are tilted by 71 and 54° ({111} and {100}) with respect to the substrate surface, such that bands in p_*t*_- and s-polarization can occur with opposite sign. The band at 2078 cm^–1^ is assigned to CO adsorbed on side facets of the NPs. The band at 2066 cm^–1^ is assigned to CO molecules adsorbed on steps and edges on side facets, as lower wavenumbers for the CO vibration correspond to adsorption on undercoordinated Pt atoms. Aside from the wavenumber, this assignment is backed up by the fact that the IR band at 2066 cm^–1^ has a lower intensity than the IR band at 2078 cm^–1^, as it was expected to observe more adsorption sites on the facets than on edges and steps as long as the NPs are large enough. A similar assignment for CO on Pt was reported in literature.^14,21^ The third IR band is negative and it is only detected in p-polarization at 2094 cm^–1^. Consequently, it can be attributed to CO adsorbed on the top facet of the NPs, excited by the p_*n*_ component of the light.

**Table 3:** FT-IRRAS Band Assignment for all Samples for Both RT and low temperature (LT)^a^

wavenumber (cm^–1^)		polarization	assignment of the CO adsorption site	
LT	RT			
	2066	p_*t*_		
2087		s	on-top Pt on side facet edges	
	2078	p_*t*_		
2095	2082	s	on-top Pt on side facets	
2101	2094	p_*n*_	on-top Pt on the top facet	Pt
1922		p_*t*_, s	Pd bridge site on side facets	
1954	1904	p_*t*_, s	Pd bridge site at defects/edges	
2040		p_*t*_, s	on-top Pt on side facet edges	
2075	2060	p_*t*_, s	on-top Pt on side facets	
2094	2086	p_*n*_	on-top Pt on the top facet	Pd_3_Pt_4_
1820	1830	p_*t*_	Pd hollow site on side facets	
1850	1860	p_*t*_	Pd hollow site on side facets	
1925	1925	p_*t*_, s	Pd bridge site on side facets	
1949	1943	p_*t*_, s	Pd bridge site at defects/edges	
	2051	p_*t*_, s	on-top Pt on side facet edges	
2079	2065	p_*t*_, s	on-top Pt on side facets	
	2084	p_*n*_	on-top Pt on the top facet	
2124		p_*t*_		PdPt
	1825	p_*t*_, s	Pd hollow site on side facets	
	1852	p_*t*_, s	Pd hollow site on side facets	
	1910	p_*t*_, s	Pd bridge site on side facets	
1942	1935	p_*t*_, s	Pd bridge site at defects/edges	
2066	2044	p_*t*_, s	on-top Pt on side facet edges	
2083	2060	p_*t*_, s	on-top Pt on side facets	
	2075	p_*n*_	on-top Pt on the top facet	Pd_2_Pt
1865	1830	p_*t*_, s	Pd hollow site on side facets	
1890	1860	p_*t*_, s	Pd hollow site on side facets	
1966	1925	p_*t*_, s	Pd bridge site on side facets	
1994	1950	p_*t*_, s	Pd bridge site on side facet edges	
	2045	p_*t*_	on-top Pt site on side facet edges	
2085	2064	p_*t*_, s	on-top Pt site on side facets	
2102		p_*t*_	on-top site on Pd on side facet	Pd_3_Pt
	1840–1850	p_*t*_, s	Pd hollow site on side facets	
1889	1870	p_*t*_, s	Pd hollow site on side facets	
1956	1915–1930	p_*t*_, s	Pd bridge site on side facets	
1985	1950	p_*t*_, s	Pd bridge site at defects/edges	
2102		p_*t*_, s	on-top site on Pd on side facet	
2130		p_*t*_, s		Pd

a The wavenumbers correspond to the CO bands at CO saturation coverage.

On pure Pd NPs on the other hand, four different bands can be identified in Figure 3, all in s-as well as p-polarization, all excited by either the p_*t*_ or s component of the light. In the wavenumber range of CO adsorption on bridge sites, two bands are visible at 1930 cm^–1^ and at 1950 cm^–1^. The IR band at 1930 cm^–1^ band is in good agreement with literature values for CO adsorption at bridge sites on Pd NP facets or single crystals. The IR band at a higher wavenumber (1950 cm^–1^) may be assigned to CO adsorbed on Pd(100) bridge sites.^33^ But even though it is expected that these types of facets exist, a different possible interpretation is that the IR band can be assigned to the CO at bridge sites on edges of the NPs, as it was reported in literature.^10,15,32,52^ The latter assignment will be adopted here, in line with the pure platinum sample, where edge and facet adsorption could also be distinguished. A very broad IR band is visible at around 1860 cm^–1^ in the wavenumber range of CO on hollow sites. It is assumed here to be constituted of two overlapping bands, at ∼1850 and at ∼1870 cm^–1^. It was reported that CO on Pd can adsorb in different adlayer configurations at hollow sites that result in two IR bands.^33^ As the specific structure is unclear, they will be named blue-shifted and red-shifted hollow site bands.

**Figure 4:**
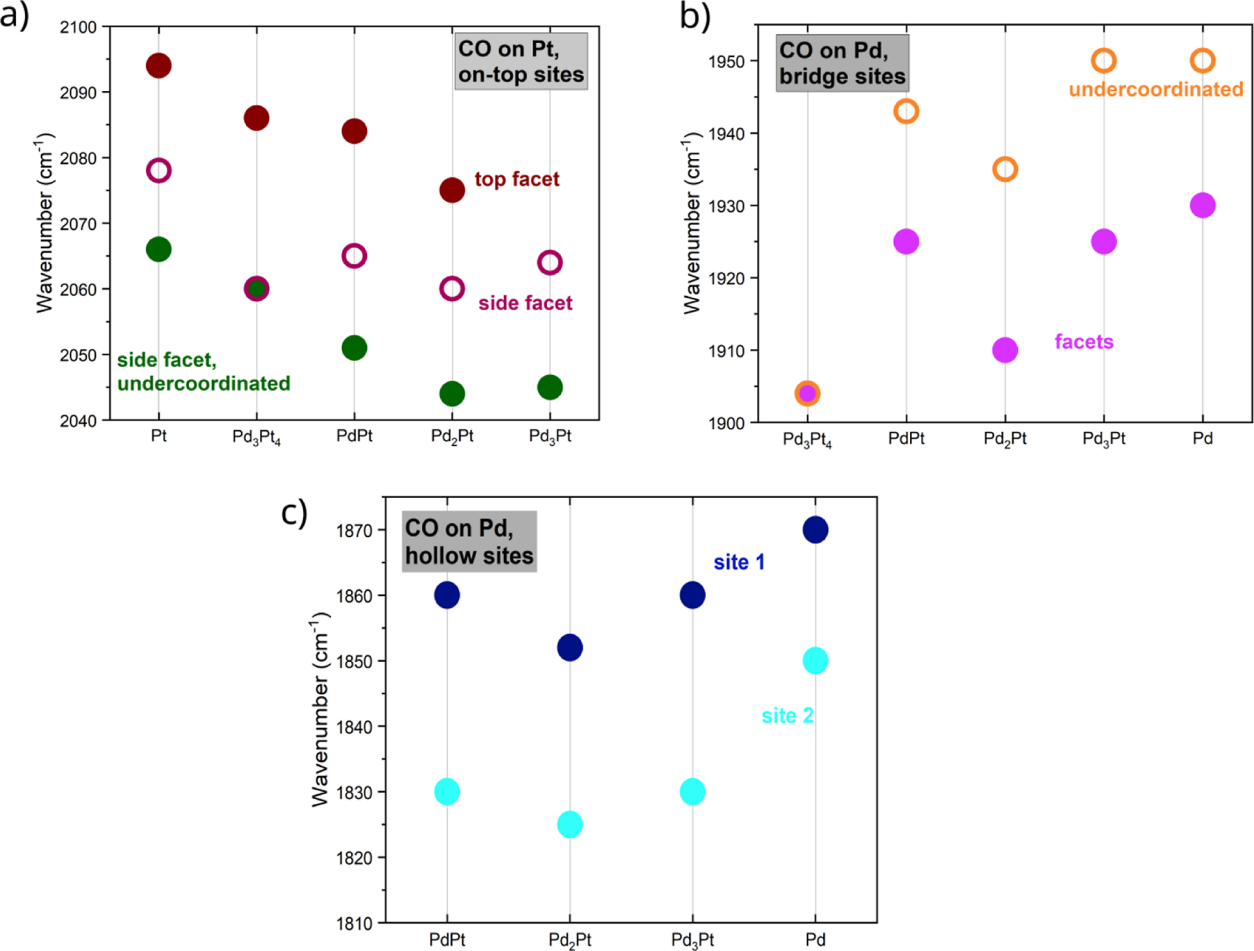
Comparison of the wavenumbers assigned to CO on (a) on-top sites on Pt, (b) bridge sites on Pd and (c) hollow sites on Pd. The data is taken from measurements at RT at p-polarization after exposure to 5 L CO (dosed at 10^–8^ mbar partial pressure).

Next, we will discuss the alloy composition dependence of the IR bands. The IR bands observed for Pd/Pt alloy NPs are a combination of the bands for Pd and Pt metal NPs, no additional new bands were observed. However, the bands are shifted for different alloy compositions and the individual intensities vary. Figure 4 gives an overview of the shifts of the bands due to alloy composition.

The IR bands corresponding to CO on Pd hollow sites (1820–1870 cm^–1^) are detected for NP alloy compositions up to PdPt. Hence, as long as a sufficient amount of palladium is present at the surface, both hollow site adsorption types exist at RT. One band is at a higher wavenumber around ∼1860 cm^–1^ and one at a lower wavenumber around ∼1830 cm^–1^. The position of the IR bands corresponding to adsorption on hollow sites stays constant as a function of the alloy composition, aside from a slight red shift when going from pure Pd NPs to an alloy, see Figure 4. From the DFT-calculations one can see a slight increase of adsorption energy of hollow sites with increasing Pd/Pt ratio in the slab, while the surface or subsurface composition exhibits a less straightforward effect on CO-binding. Thus, the red shift can be attributed to the higher amount of CO on Pd hollow sites in pure palladium NPs, and therefore the higher amount of repulsive interaction between the adsorbed CO molecules. The IR bands corresponding to CO adsorbed on Pd bridge sites show a slight blue shift for higher amounts of Pd in the alloy (Figure 4). For Pd_3_Pt_4_ the intensity of the IR band corresponding to an on-top adsorption of CO on Pt at 2060 cm^–1^ is significantly stronger than the intensity of the band corresponding to adsorption on Pd bridge sites at 1904 cm^–1^. For this alloy composition, theory predicts similar adsorption energies for both sites, see Figure S3 in the Supporting Information. We argue that the strong suppression of the bridge site signal can be taken as an indication of Pt surface segregation for Pd_3_Pt_4_. Both IR bands from CO at bridge sites of Pd on NP facets (around 1920 cm^–1^) and edges (around 1915 cm^–1^) are present for all NP alloy compositions. The wavenumber of these bands is seen to be almost constant for the samples with Pd excess, whereas it decreases for Pd_3_Pt_4_ to 1904 cm^–1^ while both bands coalesce into a single band. Hence, as long as there is more Pd than Pt in the alloy samples, the surface of the particles contains enough Pd atoms to allow for similar adsorption sites for CO as the pure Pd sample does. Pt excess in the alloy changes this, resulting in significantly less adsorption sites on Pd atoms.

The three CO bands corresponding to on-top adsorption on Pt [for side facet (2078 cm^–1^), side facet edges (2066 cm^–1^) and top facet (2094 cm^–1^)] red shift for a decreasing amount of platinum in the alloy samples (see Figure 4). The red shift of the CO band for an increasing amount of Pd can be attributed to the change of the electronic structure of the adsorption sites (more Pd), see theory section. A different explanation could be that with an increase of Pd, the Pt atoms are pushed toward the edges of the NPs. This would explain why the band corresponding to adsorption on side facets on pure Pt (2078 cm^–1^) shifts toward the position of the band corresponding to adsorption on edges on pure Pt (2066 cm^–1^). Notably, the IR band corresponding to CO on-top at the top facet is not observed for the alloy samples with a high amount of Pd. An explanation for this could be that the top facet for NPs with high amounts of Pd is smaller and rounder particle shapes are favored. This is backed up by the fact that the NPs with high amounts of Pd in the alloy composition have a higher aspect ratio height/diameter than the NPs without Pd, see Table 1.^6^ Regarding the order in which the IR bands arise (which can be found in the Supporting Information, Figures S13–S24), it is observed that the adsorption starts at the lowest wavenumber IR bands available for Pd and Pt sites separately. This suggests that CO at low coverages adsorbs on Pt on NP edges and on Pd on NP facets. Adsorption sites on Pt facets and on Pd edges are occupied only at higher dosages of CO.

**Figure 5:**
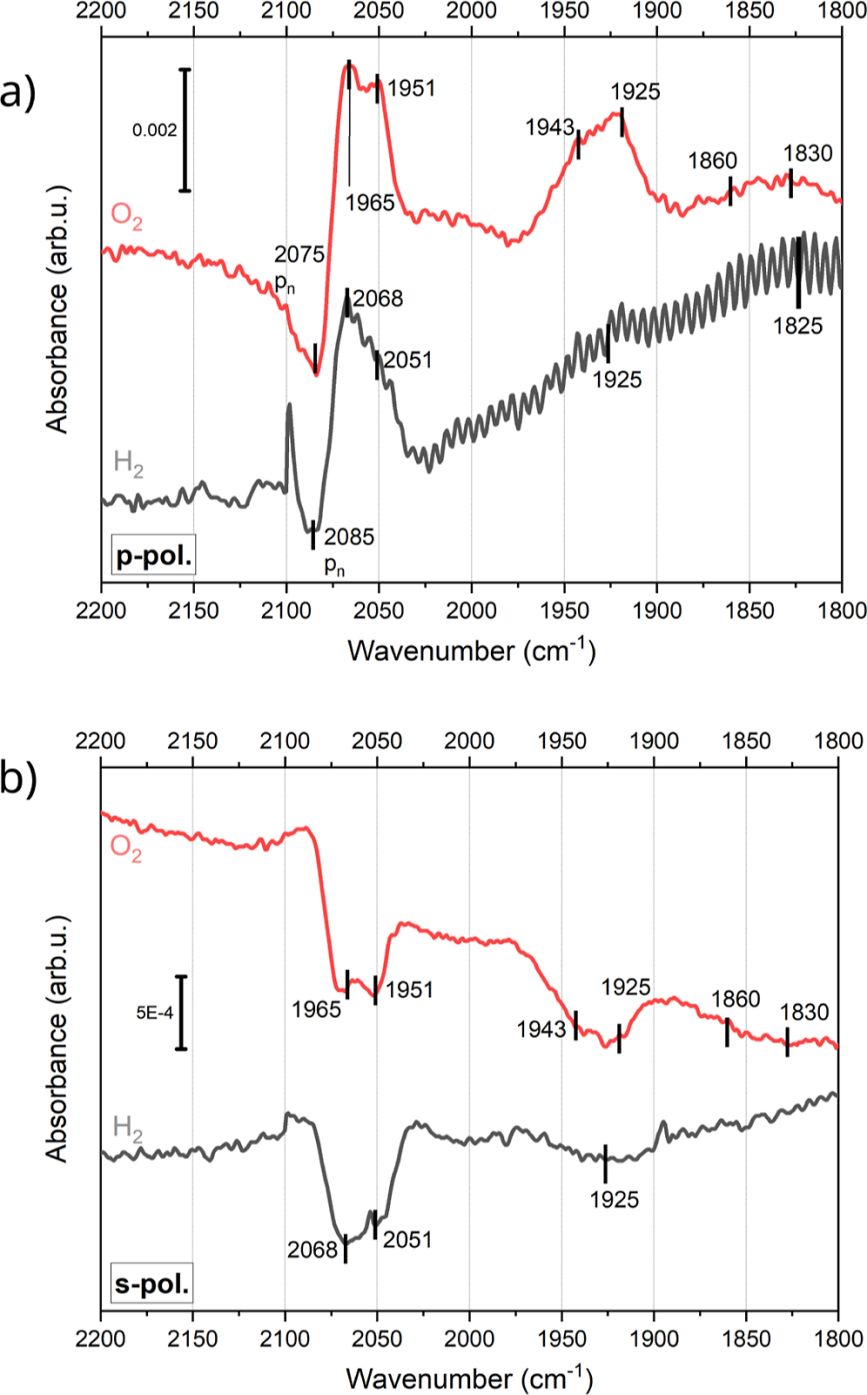
Comparison of the CO adsorption on the PdPt sample annealed in hydrogen (black) and annealed in oxygen (red) at RT after dosing 5 L CO (a CO partial pressure of about 10^–8^ mbar was used). (a) is p-polarized, (b) is s-polarized. In (a), signals that originate from the p_*n*_ part of the light are labeled.

To investigate the behavior of the alloy system under higher CO coverages, the measurements were repeated at low temperature (LT) (−130 to −160 °C). Moreover, adsorption of CO on a bare α-Al_2_O_3_ substrate was probed at LT, yielding no observable IR band (see Figure S12 in the Supporting Information) and thus excluding effects of the substrate. The results of 5 L CO adsorption on pure metal and alloy NPs at LT can be found in the Supporting Information Figure S26. All trends found at LT are the same as at RT, but the bands are blue-shifted due to a higher CO coverage. Furthermore, two additional bands appear: At 2102 cm^–1^ a band assigned to CO on-top Pd can be found on pure Pd NPs and on the Pd_3_Pt alloy NPs, the origin of the other band at 2130 cm^–1^ is unclear. Furthermore, the PdPt sample was annealed in 10^–6^ mbar H_2_ for 30 min at 670 K to investigate the effect of different gas treatments on the surface composition. The dramatic changes in the CO adsorption at RT can be seen in Figure 5, the band assignment is available in Table 4 and the full data set in the Supporting Information (Figure S25). At RT, the IR bands from CO adsorbed at on-top sites of facets and edges of Pt are visible at 2051 and 2068 cm^–1^, respectively. The p_*n*_-activated IR band assigned to CO adsorbed on on-top sites on Pt at the top facet is found at 2085 cm^–1^. From the bands corresponding to CO adsorbed on bridge sites on Pd, only a single weak p_*t*_-activated band is visible at 1925 cm^–1^. Similarly, only one weak IR band attributed to CO adsorbed on Pd on hollow sites is visible at 1825 cm^–1^. These changes in the CO adsorption after hydrogen annealing suggest that the surface is covered mostly by Pt atoms.

**Table 4:** FT-IRRAS Band Assignment of the Data from the PdPt Sample after Annealing in Hydrogen

wavenumber (cm^–1^)	polarization	assignment of the CO adsorption sit
1825	p_*t*_	Pd hollow site on side facets/edges
1925	p_*t*_, s	Pd bridge site at facets/edges
2051	p_*t*_, s	on-top Pt on side facet edges
2068	p_*t*_, s	on-top Pt on side facets
2085	p_*n*_	on-top Pt on the top facet

### Comparison of Theory and Experiment

To analyze the effect of the surface composition on the vibrational frequencies of adsorbed CO, several structural models were investigated using DFT calculations. Due to anharmonicity and the systematic DFT error, it should not be expected that the absolute value of the computed frequency of CO would fit well with the experimental data. Thus, to eliminate systematic errors and to compare with experiment, we followed the common approach to shift the computed vibrational frequencies by a constant factor (shown in Table S1) so that the computed gas phase vibration matches the experimental value (2143.0 cm^–1^).^53^ The same shift is applied to all computed vibrations of adsorbed species calculated by the same setting.

**Figure 6:**
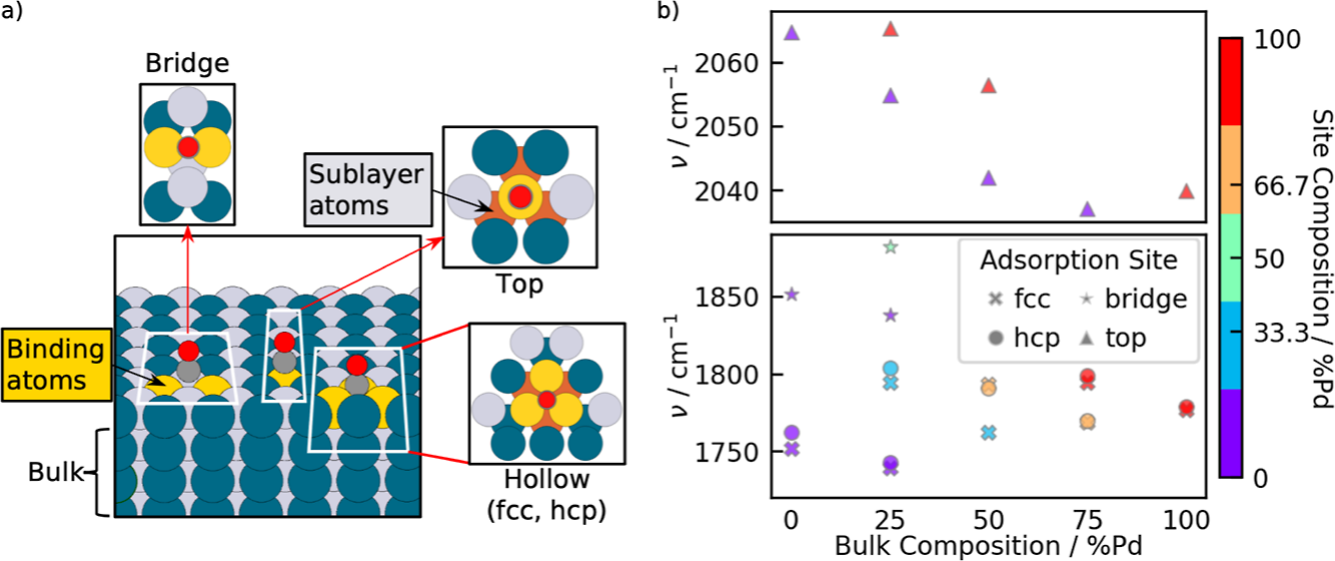
(a) Illustration of the CO adsorption sites on the fcc(111) surface based on a bulk alloy with 50% Pd in the Pd/Pt alloy system. The binding atom at the active site are highlighted in yellow and the subsurface layer is indicated in red. Pd and Pt are shown in blue and light gray, C and O in dark gray and red. (b) CO vibrational frequencies adsorbed on different sites on (111) surfaces with varying compositions. All surfaces are assumed to have a stoichiometric termination of ordered bulk alloys.

We studied adsorption on the fcc(111) facets and systematically varied the composition of the binding site and the next-nearest neighbors in the sublayer, as shown in Figure 6a. Adsorption was considered at on-top, bridge and hollow sites. In contrast to the experiment, here a distinction was made between fcc and hcp hollow sites. Both sites are located between three atoms, but the sublayers are different due to the fcc stacking. As illustrated in Figure 6a, we define the site as consisting of the binding metal atoms, i.e. one/two/three atoms for top/bridge/fcc + hcp sites. The atoms, which were varied in the sublayer are also indicated. An overview of the varied compositions can be seen in Table 5.

**Table 5:** Studied Compositions for the Different Sites, Atoms in the Sublayer and for the Bulk Composition, See Also Figures 6 and 7

category	% Pd
site: top	0, 100
site: bridge	0, 50, 100
site: hollow (fcc, hcp)	0, 33.3, 66.7, 100
sublayer	0, 33.3, 66.7, 100
bulk	0, 25, 50, 75, 100

As a first step, we have studied the fcc(111) surfaces that result from stoichiometric terminations of pure metals (Pd and Pt) and bulk alloys (L1_0_ and L1_2_) with the composition (PtPd_3_, Pt_2_Pd_2_, Pt_3_Pd, see Figure S1). These surfaces already give rise to many possible compositions of active sites and sublayers, as shown in Figure 6b. Figure 6b shows the computed harmonic frequency of adsorbed CO as a function of the bulk composition of the slab. Additionally, the composition of the binding site is illustrated with a color code. The general observation is that—for a constant composition of the active site—the vibrational frequencies decrease with an increasing amount of Pd in the bulk. One can furthermore see that the trend with respect to active site composition is opposite to that of bulk composition: the frequency generally increases with increasing amounts of Pd in the nearby atoms. The frequencies for on-top binding are in the range of 2040–2060 cm^–1^ and are lower for increasing Pd-content in the bulk. The frequencies for CO binding on top of Pt or Pd are similar, although we note that our DFT calculations predict CO-binding on top of Pd not to be favorable (see Figure S3), in agreement with the experimental observations. Frequencies for binding at hcp and fcc sites are very similar (1740–1800 cm^–1^), although our calculation also shows the well-known preference for binding at the fcc-site (see Figure S3). Bridge-sites were generally not found to be particularly stable, but the predicted frequencies agree with the observed experimental trend, i.e. they are found at higher frequencies than fcc, at around 1850 cm^–1^.

**Figure 7:**
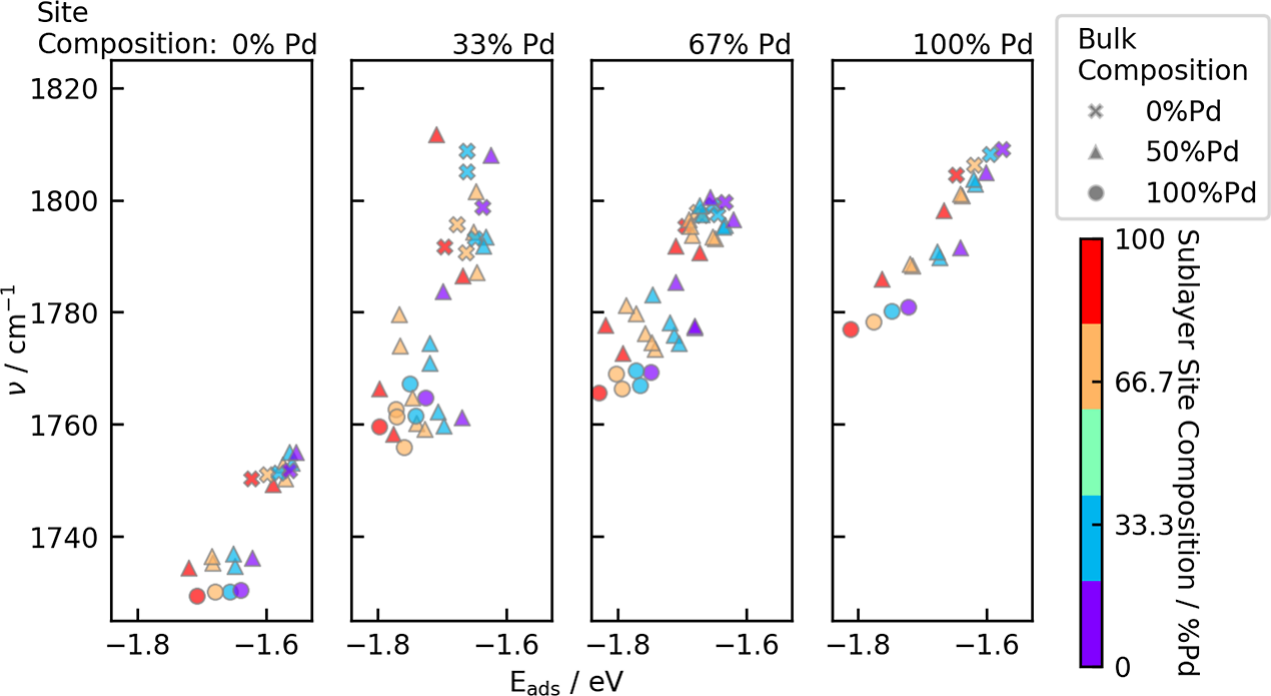
CO vibrational frequencies on fcc sites are shown as a function of the adsorption energies. Each panel contains structures with one site composition. The bulk composition is indicated with different symbols and the composition of the sublayer can be identified from the color of the symbols.

For adsorption at the fcc site, the influence of the closest atoms was further analyzed through variation of the three binding atoms and the three sublayer atoms in the possible compositions (Pt_3_, Pt_2_Pd, PtPd_2_, Pd_3_). These sites were created by substituting the three atoms in the fcc site as well as the three subsurface atoms in the stoichiometric surfaces mentioned above (bulk compositions Pd, PtPd_3_, Pt_2_Pd_2_, Pt_3_Pd and Pt). Figure 7 shows the computed data as a function of three parameters: bulk composition, site composition and sublayer composition. In agreement with trends observed for the stoichiometric, ordered surfaces, CO-frequencies generally increase with Pd-content in the fcc site. On the other hand, frequencies decrease with increasing Pd content in the bulk. The influence of the sublayer composition on the vibrational frequency is insignificant compared to the other factors.

In contrast to the theory, in the experiment only the overall composition of the particles is known and not how it relates to the exact composition of the binding site. The trend observable in Figure 4a for CO bound on top of Pt atoms is in agreement with the trend we find in Figure 6b. For the other binding sites (Figure 4b,c), there is no clear trend in the experimentally observed frequencies with particle composition. Based on our calculations, this can be explained by the competing influences of changes in the composition of both the surface site and the bulk.

The DFT calculations offer insights into the dependence of the vibrational bands on the specific local alloy composition, whereas the FT-IRRAS results yield information on the sum of the wavenumbers of all adsorption sites. The DFT calculations for hollow site adsorption of CO show a red shift for increasing amounts of nearby Pt atoms. A similar trend can be seen in the FT-IRRAS measurement for the adsorption of CO on bridge sites, see Figure 4. For on-top adsorption sites, however, the experimental results show the opposite behavior, see Figure 4. The very broad IR bands measured for the alloy samples can be related to the different specific adsorption configurations, as the DFT calculations showed that IR band wavenumber shifts several 10 cm^–1^ for different sublayer and nearby atom configurations. This can best be seen for on-top adsorption of CO on Pt on side facets (2078 cm^–1^ for pure Pt NPs): The band is strongly broadened for alloy NPs, featuring a long shoulder toward lower wavenumbers.

## Conclusions

In this work we investigated the adsorption of CO on Pd, Pt, and Pd/Pt alloy NPs on α-Al_2_O_3_. All NPs were grown on α-Al_2_O_3_ single crystal substrates by molecular beam epitaxy under UHV conditions. The resulting epitaxial NPs were exclusively (111)-oriented. Via polarization-dependent FT-IRRAS the active sites of CO adsorption on bare metal and alloy NPs were probed. Our main findings are that the adsorption sites of CO vary depending on the NP alloy composition: on pure Pt NPs, CO exclusively adsorbs in on-top configuration on single Pt atoms, whereas it mainly adsorbs on bridge sites and hollow sites between two or three atoms on pure Pd NPs. On alloy NPs both adsorption modes are found simultaneously, highlighting that the surface of alloy NPs is constituted from Pd and Pt atoms. For Pt-rich particles, however, the intensity of the adsorption bands of Pd hollow and bridge sites is significantly lower than for Pd-rich particles. This may be attributed to Pt surface segregation, in line with a theoretical study.^28^ Moreover, adsorption of CO on the top facet was only found to appear on Pt atoms, suggesting that the Pd-containing particles feature smaller top facets, in line with the shape determined by XRD. The effect of a reducing treatment on the alloy NPs was studied by annealing the samples in a hydrogen atmosphere. The CO adsorption on this reduced sample showed that the surface is most likely covered by a shell of Pt atoms. This demonstrates that the surface chemistry of alloy NPs can be tuned by appropriate gas treatments. To confirm the IR band assignments, DFT calculations were performed simulating the adsorption of CO on different Pd/Pt alloy compositions. From the DFT calculations, the conclusion can be drawn that the existence of different nearby alloy compositions for CO adsorption sites leads to broad IR bands due to different wavenumbers for different specific configurations. The results from this study help to better understand the active sites on Pd/Pt alloy catalysts and thus to produce and develop more effective catalysts for heterogeneous catalysis applications in emission control and energy conversion reactions. The use of polarization dependent infrared spectroscopy in combination with CO as a probe molecule enabled the identification of the composition-dependent adsorption on e top and side facets of the NPs, highlighting that this method will be beneficial for further studies on alloy NPs under reaction conditions where CO is a product or educt.

## Materials and Methods

All measurements and sample preparations were performed at DESY Nanolab.^54^ The Pt, Pd and Pt/Pd NPs were grown in UHV on sapphire α-Al_2_O_3_(0001) substrates via molecular beam epitaxy, the alloy particles were grown by codeposition of Pd and Pt from two flux cells of a triple electron beam evaporator. The crystal was mounted on a molybdenum sample holder with tantalum clips. Clips and sample holder were cleaned three times in an ultrasonic bath; first in acetone, then in ethanol and finally in isopropyl. The substrate was degassed at 450–650 °C for 120 min under UHV and afterward annealed in 10^–6^ mbar oxygen for 120 min at 300 °C. Pd and Pt NPs were grown via MBE at sample temperatures between 400 and 450 °C. The alloy composition was controlled by adjusting the flux of the individual metal vapors. These have been calibrated up to an error of 10% by growing NPs of only one metal atomic species. For the sample preparation and the FT-IRRAS experiments, high-purity gases [oxygen (99.999%), carbon monoxide (99.97%), hydrogen (99.9999%)] were used. SEM images were obtained in secondary electron mode with a field emission instrument at an accelerator voltage of 5 kV, providing a nominal lateral resolution of 1.3 nm. Grazing incidence XRD and reflectivity measurements were performed at a photon energy of 8.04 keV (Cu Kα radiation) using a hybrid pixel detector with a GaAs sensor employing a six-circle diffractometer setup.

FT-IRRAS measurements were performed with a commercial spectrometer in combination with a liquid nitrogen cooled mercury–cadmium–telluride (MCT) detector and a UHV sample chamber. The resolution of the setup is 0.2 cm^–1^, the incidence angle is 85° with respect to the surface normal. For LT measurements, the sample station of the spectrometer could be cooled down to −160 °C (113 K). In the following it will be distinguished between RT and LT, which corresponds to liquid nitrogen cooling [corresponding to temperatures between −130 and −160 °C (140 and 110 K)].

In-between CO adsorption measurements, the sample had to be cleaned. This was done by annealing in 10^–6^ mbar oxygen at 250 °C for 30 min and then flashing to 400 °C (still in oxygen). Note that under these conditions PdO surface oxides are not stable.^55^ The samples were cooled down to RT under UHV conditions after O_2_ was pumped out of the system, removing also residual chemisorbed oxygen. For one sample the effect of hydrogen annealing was tested by not cooling down the sample after the flash annealing, but leaving it at 400 °C under UHV for 20 min and then under 10^–6^ mbar H_2_ for 30 min, followed by cooldown under UHV.

All DFT calculations were performed with the Vienna Ab-initio Simulation Package (VASP) version 5.4.1^56,57^ and the atomic simulation environment^58^ using the BEEF-vdW functional^59,60^ and the projector-augmented wave method.^61,62^ The lattice constants of Pd/Pt alloys were optimized using an energy cutoff of 600 eV, while slabs calculations were performed with a cutoff of 400 eV and Γ-centered *k*-point grid with a 6 × 6 × 1 mesh for (2 × 2) surface slab unit. Surfaces were modeled with a four-layer slab, with two bottom layers frozen. To eliminate artificial interactions due to periodic boundary conditions, the slabs are separated by 28 Å of vacuum. The unit cells of the alloys and their calculated lattice constant are shown in Figure S1. CO-adsorption was studied in the low-coverage limit (θ = 1/16). The vibrational frequencies of adsorbed CO were calculated within the four-point harmonic approximation. More information about DFT methodology can be found in Supporting Information.

## Supporting Information Available

Additional figures; the full data sets of the FT-IRRAS measurements; the data of the XRD, XRR and SEM measurements; the cleaning and annealing procedure; more Details about the computational method (PDF)

## Notes

An early version of this work has been published on a preprint server: Dolling DS, Chen J, Schober J-C, Creutzburg M, Jeromin A, Vonk V, et al. Probing active sites on Pd/Pt alloy nanoparticles by CO adsorption; 2024; ChemRxiv; doi: 10.26434/chemrxiv-2024-c3hwg;https://chemrxiv.org/engage/chemrxiv/article-details/66016c499138d231613f54ba (accessed October 08, 2024).
