# Supplementary material for: Probing Active Sites on Pd/Pt Alloy Nanoparticles by CO Adsorption
Source: ACS Nano. 2024 Nov 2;18(45):31098–108. doi: 10.1021/acsnano.4c08291 (PMC11562784; doi:10.1021/acsnano.4c08291)
Supplement: Supplementary file 1 — nn4c08291_si_001.pdf [file nn4c08291_si_001.pdf]

Supporting Information – Probing active sites on Pd/Pt alloy nanoparticles by CO adsorption

Daniel Silvan Dolling,[†,‡] Jiachen Chen,[¶] Jan-Christian Schober,[†,‡] Marcus Creutzburg,[†] Arno Jeromin,[†] Vedran Vonk,[†] Dmitry I. Sharapa,[¶] Thomas F. Keller,[†,‡] Philipp N. Plessow,[¶] Heshmat Noei,[*,†,‡] and Andreas Stierle[*,†,‡]

†*Centre for X-ray and Nano Science CXNS, Deutsches Elektronen-Synchrotron DESY, 22607 Hamburg, Germany*

‡*Fachbereich Physik, Universität Hamburg, 20355 Hamburg, Germany*

¶*Institute of Catalysis Research and Technology (IKFT), Karlsruhe Institute of Technology (KIT), 76344 Eggenstein-Leopoldshafen, Germany*

E-mail: heshmat.noei@desy.de; andreas.stierle@desy.de

Additional details of computational methodology

The expansion of the density includes reciprocal lattice vectors has a norm up to 3/2 times larger than for the wave function, $|\mathbf{G}_{\text{cut}}|$ (PREC=Normal in VASP). The calculations were performed with real-space projectors (LREAL=AUTO) and the self-consistent field (SCF) procedure was converged to a threshold of 10^{-8} eV for the total energy. A criterion of 0.01 eV/Å for the maximum forces on individual atoms was used for geometry convergence. Gaussian smearing with a 0.1 eV width was applied.

The unit cells of the alloys and their calculated lattice constant are shown in Fig. S1.

Only CO was included in the numerical Hessian, with a displacement of 0.02 Å.

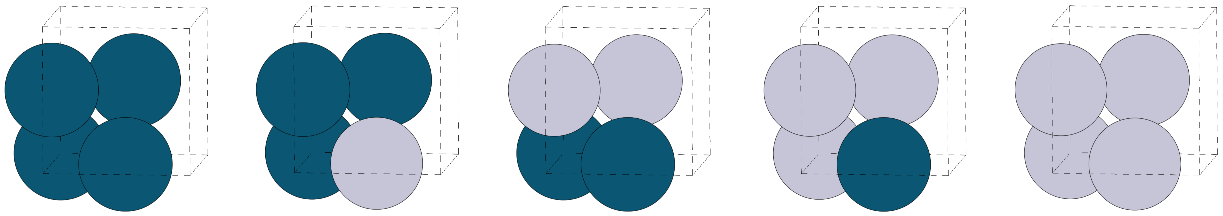

Figure S1: The unit cells of Pd/Pt alloy bulk with different compositions and their lattice parameters.

The theoretical CO-vibrational frequency is obtained by Eq. S1, shifting the computed value by the difference between gas phase values from the experiment[1] (2143.0 cm^{-1}) and DFT.

$$\nu_{\text{theory}} = \nu_{\text{DFT}} + (\nu^{\text{CO}}_{\text{DFT}} - \nu^{\text{CO}}_{\text{exp.}}) \tag{S1}$$

The DFT value varies slightly with the setting, as shown in Table S1. For the calculation of the shift of adsorbed CO, the vibration of adsorbed CO, as well as CO in the gas phase have always been computed with a consistent setting.

The vibration data are shown in Table S2, which is calculated by setting PREC = Normal and NFREE = 2, except for the top site on 100 Pd surfaces, which is PREC = Accurate and NFREE = 4. The vibration data are shown in Table S3, which is calculated by setting PREC = Normal and NFREE = 4.

Table S1: DFT value for CO-vibrational frequency in the gas phase. δ is the displacement used in the calculation of the numerical Hessian. NFREE corresponds to the number of force calculations performed per Cartesian component of an atom. The shift constant is defined as $\nu_{\mathrm{DFT}}^{\mathrm{CO}} - \nu_{\mathrm{exp.}}^{\mathrm{CO}}$.

PREC	NFREE	δ / Å	ν / cm^{-1}	Shift Constant / cm^{-1}
normal	2	0.01	2124.4	-18.6
normal	4	0.02	2122.7	-20.3
accurate	4	0.02	2122.3	-20.7

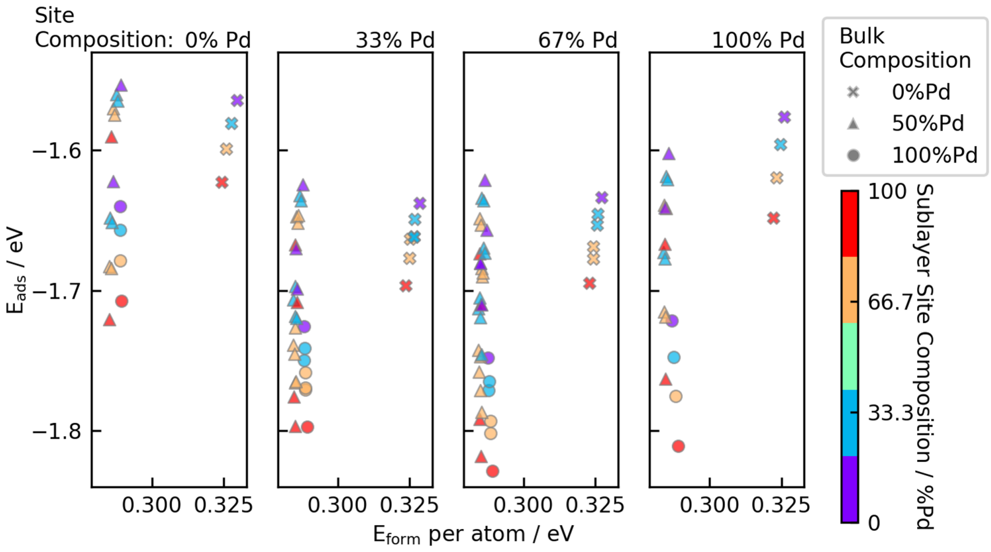

Figure S2: CO/fcc adsorption energies are shown as a function of the formation energies of slabs. Each panel contains structures with one site composition. The bulk composition is indicated with different symbols and the composition of the sublayer can be identified from the color of the symbols.

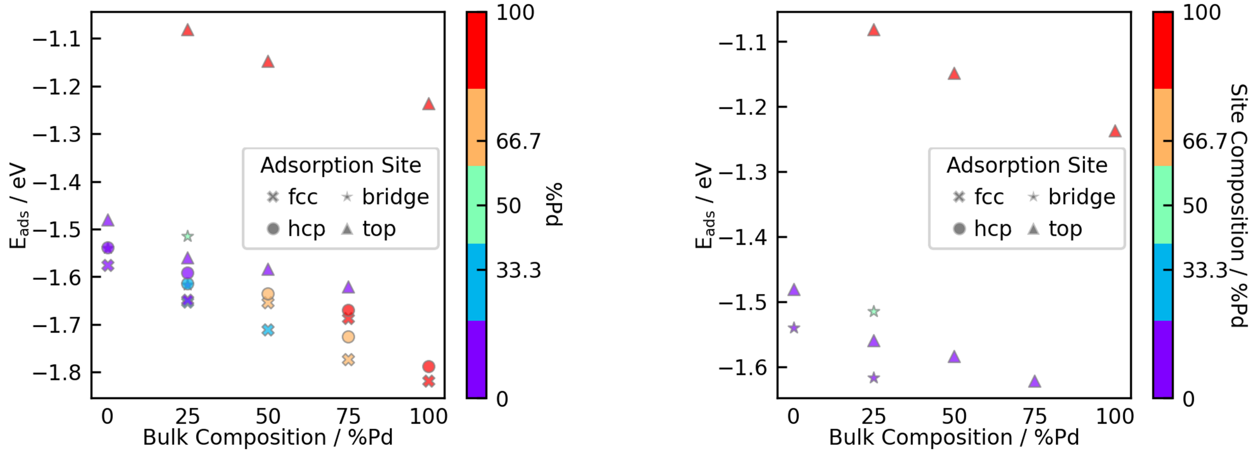

Figure S3: The adsorption energy of the same structures shown in Fig. 7. (right) only shows the top site and bridge site

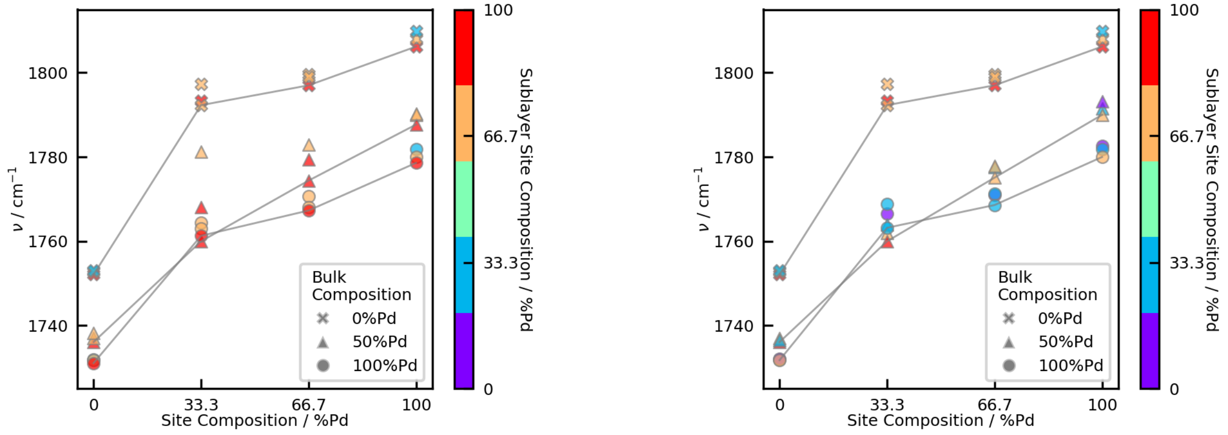

Figure S4: CO vibrational frequencies at fcc sites in different slabs. The slab of 50% Pd is shown in Fig. 8, and the three nearby and closest atoms in the sublayer are variable. Only three data points are shown for each site of each slab type with (left) the lowest adsorption energy (right) the lowest formation energy of the clean surface. Different markers indicate various bulk compositions and the x-axis represents the composition of the nearby atoms. The coloration signifies the composition of the three closest in the sublayer.

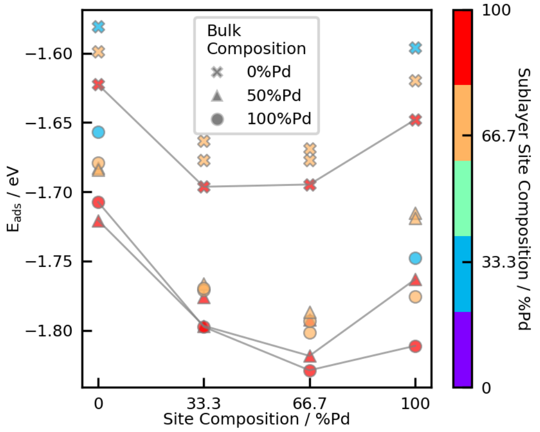

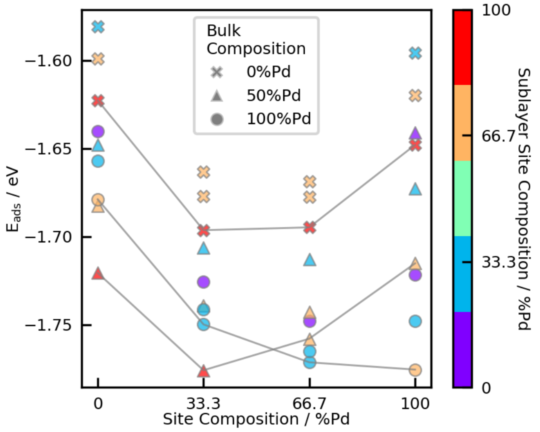

Figure S5: Adsorption energy of CO at fcc sites in different slabs. The slab of 50% Pd is shown in Fig. 8, and the three nearby and closest atoms in the sublayer are variable. Only three data points are shown for each site of each slab type with (left) the lowest adsorption energy (right) the lowest formation energy of the clean surface. Different markers indicate various bulk compositions and the x-axis represents the composition of the nearby atoms. The coloration signifies the composition of the three closest in the sublayer.

Data

In tables S2, S3 the frequencies obtained in this way are reported.

Table S2: Shifted vibrational frequencies (in cm^{-1}) are used in Fig. 7 and S3. Total energies of the clean slab and adsorption energies are also shown, both are shown in eV. All the $\theta = 1/4$ data are calculated on 2×2×4 slabs and all the $\theta = 1/16$ data are calculated on 4×4×4 slabs.

Slab Composition	E_{total}	Site	Site Composition	ν		E_{ads}	
				θ		θ	
				1/4	1/16	1/4	1/16
100% Pd	-26.560	fcc	Pd_3	1817.6	1776.8	-1.733	-1.818
		hcp	Pd_3	1816.0	1778.9	-1.729	-1.788
		top	Pd	2055.6	2038.6	-1.238	-1.236
75% Pd	-31.395	fcc	Pd_2Pt_1	1813.9	1768.7	-1.700	-1.772
		fcc	Pd_3	1833.4	1794.3	-1.587	-1.686
		hcp	Pd_2Pt_1	1809.5	1769.5	-1.674	-1.726
		hcp	Pd_3	1835.0	1798.8	-1.626	-1.67
		top	Pt	2057.2	2037.1	-1.568	-1.621
50% Pd	-36.071	fcc	Pd_1Pt_2	1811.6	1762.2	-1.647	-1.711
		fcc	Pd_2Pt_1	1831.9	1793.2	-1.569	-1.653
		hcp	Pd_1Pt_2	1823.7	1791.0	-1.585	-1.635
		top	Pt	2057.6	2042.0	-1.504	-1.584
		top	Pd	2068.3	2056.4	-1.088	-1.148
25% Pd	-40.541	fcc	Pd_1Pt_2	1852.5	1794.4	-1.554	-1.652
		fcc	Pt_3	1773.2	1739.0	-1.554	-1.648
		hcp	Pt_3	1770.4	1742.9	-1.525	-1.592
		hcp	Pd_1Pt_2	1834.6	1803.8	-1.543	-1.614
		bridge	PdPt	1901.0	1881.7	-1.447	-1.514
		bridge	Pt_2	1860.9	1838.0	-1.543	-1.617
		top	Pt	2069.5	2054.8	-1.457	-1.559
		top	Pd	2076.3	2065.3	-1.031	-1.081
0% Pd	-44.787	fcc	Pt_3	1782.1	1751.9	-1.449	-1.576
		hcp	Pt_3	1787.5	1762.0	-1.447	-1.539
		bridge	Pt_2	1874.9	1851.1	-1.457	-1.540
		top	Pt	2076.2	2064.7	-1.394	-1.480

Table S3: Shifted vibrational frequencies (in cm^{-1}) are used in Fig. 8, S2, S4 and S5. Total energies and formation energy of the slabs and adsorption energies are also shown, all are shown in eV. All the slabs are 4×4×4.

Bulk Composition	Label	Site Composition	Sublayer Composition	E_{total}	E_{form}	ν	E_{ads}
100% Pd	Pd_{64}	Pd_3	Pd_3	-120.151	18.498	1778.6	-1.811
100% Pd	$Pd_{63}Pt\_1$	Pd_2Pt_1	Pd_3	-121.339	18.508	1767.3	-1.829
100% Pd	$Pd_{63}Pt\_2$	Pd_3	Pd_2Pt_1	-121.351	18.442	1780.0	-1.775
100% Pd	$Pd_{62}Pt_2\_1$	Pd_1Pt_2	Pd_3	-122.484	18.512	1761.3	-1.797
100% Pd	$Pd_{62}Pt_2\_2$	Pd_2Pt_1	Pd_2Pt_1	-122.530	18.462	1768.1	-1.793
100% Pd	$Pd_{62}Pt_2\_3$	Pd_2Pt_1	Pd_2Pt_1	-122.541	18.459	1770.7	-1.802
100% Pd	$Pd_{62}Pt_2\_4$	Pd_3	Pd_1Pt_2	-122.552	18.394	1781.9	-1.748
100% Pd	$Pd_{61}Pt_3\_1$	Pt_3	Pd_3	-123.569	18.516	1731.1	-1.707
100% Pd	$Pd_{61}Pt_3\_2$	Pd_1Pt_2	Pd_2Pt_1	-123.675	18.474	1764.4	-1.771
100% Pd	$Pd_{61}Pt_3\_3$	Pd_2Pt_1	Pd_1Pt_2	-123.715	18.428	1768.6	-1.765
100% Pd	$Pd_{61}Pt_3\_4$	Pd_1Pt_2	Pd_2Pt_1	-123.656	18.481	1757.6	-1.759
100% Pd	$Pd_{61}Pt_3\_5$	Pd_1Pt_2	Pd_2Pt_1	-123.673	18.474	1763.0	-1.770
100% Pd	$Pd_{61}Pt_3\_6$	Pd_2Pt_1	Pd_1Pt_2	-123.727	18.422	1771.3	-1.771
100% Pd	$Pd_{61}Pt_3\_7$	Pd_3	Pt_3	-123.742	18.357	1782.6	-1.721
100% Pd	$Pd_{60}Pt_4\_1$	Pt_3	Pd_2Pt_1	-124.742	18.494	1731.8	-1.679
100% Pd	$Pd_{60}Pt_4\_2$	Pd_1Pt_2	Pd_1Pt_2	-124.861	18.447	1768.9	-1.750
100% Pd	$Pd_{60}Pt_4\_3$	Pd_1Pt_2	Pd_1Pt_2	-124.842	18.457	1763.2	-1.741
100% Pd	$Pd_{60}Pt_4\_4$	Pd_2Pt_1	Pt_3	-124.905	18.401	1771.0	-1.748
100% Pd	$Pd_{59}Pt_5\_1$	Pt_3	Pd_1Pt_2	-125.909	18.485	1731.9	-1.657
100% Pd	$Pd_{59}Pt_5\_2$	Pd_1Pt_2	Pt_3	-126.018	18.445	1766.5	-1.726
100% Pd	$Pd_{58}Pt_6$	Pt_3	Pt_3	-127.065	18.492	1732.1	-1.640
50% Pd	$Pd_{36}Pt_{28}$	Pd_3	Pd_3	-153.424	18.215	1787.7	-1.763

Bulk Composition	Label	Site Composition	Sublayer Composition	E_{total}	E_{form}	ν	E_{ads}
50% Pd	$Pd_{35}Pt_{29}$-1	Pd_2Pt_1	Pd_3	-154.632	18.216	1774.4	-1.792
50% Pd	$Pd_{35}Pt_{29}$-2	Pd_2Pt_1	Pd_3	-154.629	18.245	1779.4	-1.818
50% Pd	$Pd_{35}Pt_{29}$-3	Pd_3	Pd_2Pt_1	-154.553	18.221	1790.3	-1.719
50% Pd	$Pd_{35}Pt_{29}$-4	Pd_3	Pd_2Pt_1	-154.582	18.189	1790.0	-1.715
50% Pd	$Pd_{34}Pt_{30}$-1	Pd_1Pt_2	Pd_3	-155.791	18.220	1760.0	-1.776
50% Pd	$Pd_{34}Pt_{30}$-2	Pd_2Pt_1	Pd_2Pt_1	-155.750	18.232	1776.3	-1.747
50% Pd	$Pd_{34}Pt_{30}$-3	Pd_2Pt_1	Pd_2Pt_1	-155.791	18.202	1777.9	-1.758
50% Pd	$Pd_{34}Pt_{30}$-4	Pd_2Pt_1	Pd_2Pt_1	-155.784	18.195	1775.1	-1.743
50% Pd	$Pd_{34}Pt_{30}$-5	Pd_1Pt_2	Pd_3	-155.790	18.242	1768.1	-1.797
50% Pd	$Pd_{34}Pt_{30}$-6	Pd_2Pt_1	Pd_2Pt_1	-155.761	18.261	1783.0	-1.787
50% Pd	$Pd_{34}Pt_{30}$-7	Pd_2Pt_1	Pd_2Pt_1	-155.776	18.231	1781.4	-1.771
50% Pd	$Pd_{34}Pt_{30}$-8	Pd_3	Pd_1Pt_2	-155.710	18.203	1792.5	-1.677
50% Pd	$Pd_{34}Pt_{30}$-9	Pd_3	Pd_1Pt_2	-155.735	18.173	1791.5	-1.673
50% Pd	$Pd_{34}Pt_{30}$-10	Pd_3	Pd_3	-155.703	18.199	1799.8	-1.667
50% Pd	$Pd_{33}Pt_{31}$-1	Pt_3	Pd_3	-156.889	18.247	1736.1	-1.721
50% Pd	$Pd_{33}Pt_{31}$-2	Pd_1Pt_2	Pd_2Pt_1	-156.894	18.248	1760.9	-1.727
50% Pd	$Pd_{33}Pt_{31}$-3	Pd_1Pt_2	Pd_2Pt_1	-156.945	18.210	1761.9	-1.739
50% Pd	$Pd_{33}Pt_{31}$-4	Pd_1Pt_2	Pd_2Pt_1	-156.913	18.268	1775.7	-1.765
50% Pd	$Pd_{33}Pt_{31}$-5	Pd_2Pt_1	Pd_1Pt_2	-156.908	18.227	1779.8	-1.719
50% Pd	$Pd_{33}Pt_{31}$-6	Pd_1Pt_2	Pd_2Pt_1	-156.926	18.235	1766.5	-1.745
50% Pd	$Pd_{33}Pt_{31}$-7	Pd_1Pt_2	Pd_2Pt_1	-156.941	18.241	1781.3	-1.766
50% Pd	$Pd_{33}Pt_{31}$-8	Pd_2Pt_1	Pd_1Pt_2	-156.898	18.222	1776.2	-1.705
50% Pd	$Pd_{33}Pt_{31}$-9	Pd_2Pt_1	Pd_1Pt_2	-156.937	18.191	1777.6	-1.713
50% Pd	$Pd_{33}Pt_{31}$-10	Pd_2Pt_1	Pd_1Pt_2	-156.906	18.255	1784.9	-1.746
50% Pd	$Pd_{33}Pt_{31}$-11	Pd_3	Pt_3	-156.859	18.197	1793.2	-1.641

Bulk Composition	Label	Site Composition	Sublayer Composition	E_{total}	E_{form}	ν	E_{ads}
50% Pd	$Pd_{33}Pt_{31}$_12	Pd_2Pt_1	Pd_3	-156.883	18.242	1793.5	-1.710
50% Pd	$Pd_{33}Pt_{31}$_13	Pd_2Pt_1	Pd_3	-156.879	18.210	1792.3	-1.674
50% Pd	$Pd_{33}Pt_{31}$_14	Pd_3	Pd_2Pt_1	-156.861	18.193	1802.5	-1.639
50% Pd	$Pd_{33}Pt_{31}$_15	Pd_3	Pd_2Pt_1	-156.834	18.223	1802.8	-1.641
50% Pd	$Pd_{32}Pt_{32}$_1	Pt_3	Pd_2Pt_1	-157.995	18.284	1738.2	-1.684
50% Pd	$Pd_{32}Pt_{32}$_2	Pt_3	Pd_2Pt_1	-158.026	18.252	1737.0	-1.683
50% Pd	$Pd_{32}Pt_{32}$_3	Pd_1Pt_2	Pd_1Pt_2	-158.043	18.250	1761.4	-1.697
50% Pd	$Pd_{32}Pt_{32}$_4	Pd_1Pt_2	Pd_1Pt_2	-158.091	18.211	1763.9	-1.706
50% Pd	$Pd_{32}Pt_{32}$_5	Pd_1Pt_2	Pd_1Pt_2	-158.068	18.246	1776.1	-1.719
50% Pd	$Pd_{32}Pt_{32}$_6	Pd_2Pt_1	Pt_3	-158.048	18.229	1779.3	-1.681
50% Pd	$Pd_{32}Pt_{32}$_7	Pd_1Pt_2	Pd_1Pt_2	-158.043	18.272	1772.7	-1.720
50% Pd	$Pd_{32}Pt_{32}$_8	Pd_2Pt_1	Pt_3	-158.048	18.228	1779.0	-1.680
50% Pd	$Pd_{32}Pt_{32}$_9	Pd_2Pt_1	Pt_3	-158.042	18.263	1787.0	-1.710
50% Pd	$Pd_{32}Pt_{32}$_10	Pd_1Pt_2	Pd_3	-158.021	18.283	1813.4	-1.708
50% Pd	$Pd_{32}Pt_{32}$_11	Pd_2Pt_1	Pd_2Pt_1	-157.998	18.284	1797.0	-1.687
50% Pd	$Pd_{32}Pt_{32}$_12	Pd_1Pt_2	Pd_3	-158.011	18.252	1788.3	-1.667
50% Pd	$Pd_{32}Pt_{32}$_13	Pd_2Pt_1	Pd_2Pt_1	-158.027	18.252	1795.5	-1.684
50% Pd	$Pd_{32}Pt_{32}$_14	Pd_2Pt_1	Pd_2Pt_1	-158.012	18.273	1798.3	-1.690
50% Pd	$Pd_{32}Pt_{32}$_15	Pd_2Pt_1	Pd_2Pt_1	-158.034	18.210	1794.9	-1.649
50% Pd	$Pd_{32}Pt_{32}$_16	Pd_2Pt_1	Pd_2Pt_1	-157.998	18.250	1795.2	-1.653
50% Pd	$Pd_{32}Pt_{32}$_17	Pd_3	Pd_1Pt_2	-157.985	18.229	1804.5	-1.618
50% Pd	$Pd_{32}Pt_{32}$_18	Pd_3	Pd_1Pt_2	-157.956	18.260	1805.5	-1.621
50% Pd	$Pd_{31}Pt_{33}$_1	Pt_3	Pd_1Pt_2	-159.126	18.300	1738.6	-1.652
50% Pd	$Pd_{31}Pt_{33}$_2	Pt_3	Pd_1Pt_2	-159.155	18.269	1736.5	-1.648
50% Pd	$Pd_{31}Pt_{33}$_3	Pd_1Pt_2	Pt_3	-159.182	18.263	1762.9	-1.670

Bulk Composition	Label	Site Composition	Sublayer Composition	E_{total}	E_{form}	ν	E_{ads}
50% Pd	$Pd_{31}Pt_{33}$-4	Pd_1Pt_2	Pt_3	-159.182	18.292	1785.4	-1.699
50% Pd	$Pd_{31}Pt_{33}$-5	Pt_3	Pd_3	-159.075	18.290	1751.0	-1.590
50% Pd	$Pd_{31}Pt_{33}$-6	Pd_1Pt_2	Pd_2Pt_1	-159.153	18.269	1803.2	-1.647
50% Pd	$Pd_{31}Pt_{33}$-7	Pd_1Pt_2	Pd_2Pt_1	-159.108	18.313	1788.8	-1.646
50% Pd	$Pd_{31}Pt_{33}$-8	Pd_2Pt_1	Pd_1Pt_2	-159.149	18.295	1799.1	-1.669
50% Pd	$Pd_{31}Pt_{33}$-9	Pd_2Pt_1	Pd_1Pt_2	-159.120	18.329	1800.7	-1.673
50% Pd	$Pd_{31}Pt_{33}$-10	Pd_1Pt_2	Pd_2Pt_1	-159.129	18.298	1796.1	-1.652
50% Pd	$Pd_{31}Pt_{33}$-11	Pd_2Pt_1	Pd_1Pt_2	-159.146	18.264	1797.2	-1.634
50% Pd	$Pd_{31}Pt_{33}$-12	Pd_2Pt_1	Pd_1Pt_2	-159.107	18.304	1796.9	-1.636
50% Pd	$Pd_{31}Pt_{33}$-13	Pd_3	Pt_3	-159.098	18.280	1806.7	-1.602
50% Pd	$Pd_{30}Pt_{34}$-1	Pt_3	Pt_3	-160.247	18.330	1737.9	-1.622
50% Pd	$Pd_{30}Pt_{34}$-2	Pt_3	Pd_2Pt_1	-160.197	18.328	1752.1	-1.570
50% Pd	$Pd_{30}Pt_{34}$-3	Pt_3	Pd_2Pt_1	-160.172	18.358	1754.6	-1.575
50% Pd	$Pd_{30}Pt_{34}$-4	Pd_1Pt_2	Pd_1Pt_2	-160.217	18.374	1793.5	-1.636
50% Pd	$Pd_{30}Pt_{34}$-5	Pd_1Pt_2	Pd_1Pt_2	-160.241	18.346	1795.2	-1.632
50% Pd	$Pd_{30}Pt_{34}$-6	Pd_2Pt_1	Pt_3	-160.245	18.367	1802.2	-1.657
50% Pd	$Pd_{30}Pt_{34}$-7	Pd_2Pt_1	Pt_3	-160.245	18.332	1798.3	-1.621
50% Pd	$Pd_{29}Pt_{35}$-1	Pt_3	Pd_1Pt_2	-161.285	18.410	1754.8	-1.560
50% Pd	$Pd_{29}Pt_{35}$-2	Pt_3	Pd_1Pt_2	-161.260	18.440	1756.8	-1.565
50% Pd	$Pd_{29}Pt_{35}$-3	Pd_1Pt_2	Pt_3	-161.340	18.419	1809.7	-1.624
50% Pd	$Pd_{28}Pt_{36}$	Pt_3	Pt_3	-162.360	18.508	1756.8	-1.553
0% Pd	Pd_6Pt_{58}	Pd_3	Pd_3	-186.297	20.624	1806.2	-1.648
0% Pd	Pd_5Pt_{59}-1	Pd_2Pt_1	Pd_3	-187.478	20.670	1797.0	-1.695
0% Pd	Pd_5Pt_{59}-2	Pd_3	Pd_2Pt_1	-187.378	20.694	1808.0	-1.620
0% Pd	Pd_4Pt_{60}-1	Pd_1Pt_2	Pd_3	-188.617	20.712	1793.4	-1.696

Bulk Composition	Label	Site Composition	Sublayer Composition	E_{total}	E_{form}	ν	E_{ads}
0% Pd	Pd_4Pt_{60}_2	Pd_2Pt_1	Pd_2Pt_1	-188.562	20.748	1799.6	-1.677
0% Pd	Pd_4Pt_{60}_3	Pd_2Pt_1	Pd_2Pt_1	-188.549	20.753	1799.0	-1.669
0% Pd	Pd_4Pt_{60}_4	Pd_3	Pd_1Pt_2	-188.456	20.773	1809.8	-1.596
0% Pd	Pd_3Pt_{61}_1	Pt_3	Pd_3	-189.685	20.751	1752.1	-1.623
0% Pd	Pd_3Pt_{61}_2	Pd_1Pt_2	Pd_2Pt_1	-189.687	20.803	1797.3	-1.677
0% Pd	Pd_3Pt_{61}_3	Pd_1Pt_2	Pd_2Pt_1	-189.667	20.809	1792.3	-1.663
0% Pd	Pd_3Pt_{61}_4	Pd_2Pt_1	Pd_1Pt_2	-189.626	20.840	1800.7	-1.653
0% Pd	Pd_3Pt_{61}_5	Pd_2Pt_1	Pd_1Pt_2	-189.612	20.846	1799.2	-1.645
0% Pd	Pd_3Pt_{61}_6	Pd_3	Pt_3	-189.527	20.862	1810.7	-1.576
0% Pd	Pd_2Pt_{62}_1	Pt_3	Pd_2Pt_1	-190.736	20.856	1752.7	-1.599
0% Pd	Pd_2Pt_{62}_2	Pd_1Pt_2	Pd_1Pt_2	-190.752	20.903	1806.8	-1.662
0% Pd	Pd_2Pt_{62}_3	Pd_1Pt_2	Pd_1Pt_2	-190.731	20.911	1794.9	-1.649
0% Pd	Pd_2Pt_{62}_4	Pd_1Pt_2	Pd_1Pt_2	-190.752	20.902	1810.4	-1.662
0% Pd	Pd_2Pt_{62}_5	Pd_2Pt_1	Pt_3	-190.683	20.943	1801.3	-1.633
0% Pd	$PdPt_{63}$_1	Pt_3	Pd_1Pt_2	-191.781	20.973	1753.1	-1.581
0% Pd	$PdPt_{63}$_2	Pd_1Pt_2	Pt_3	-191.788	21.023	1800.5	-1.637
0% Pd	Pt_{64}	Pt_3	Pt_3	-192.817	21.100	1753.5	-1.564

SEM and XRD Results

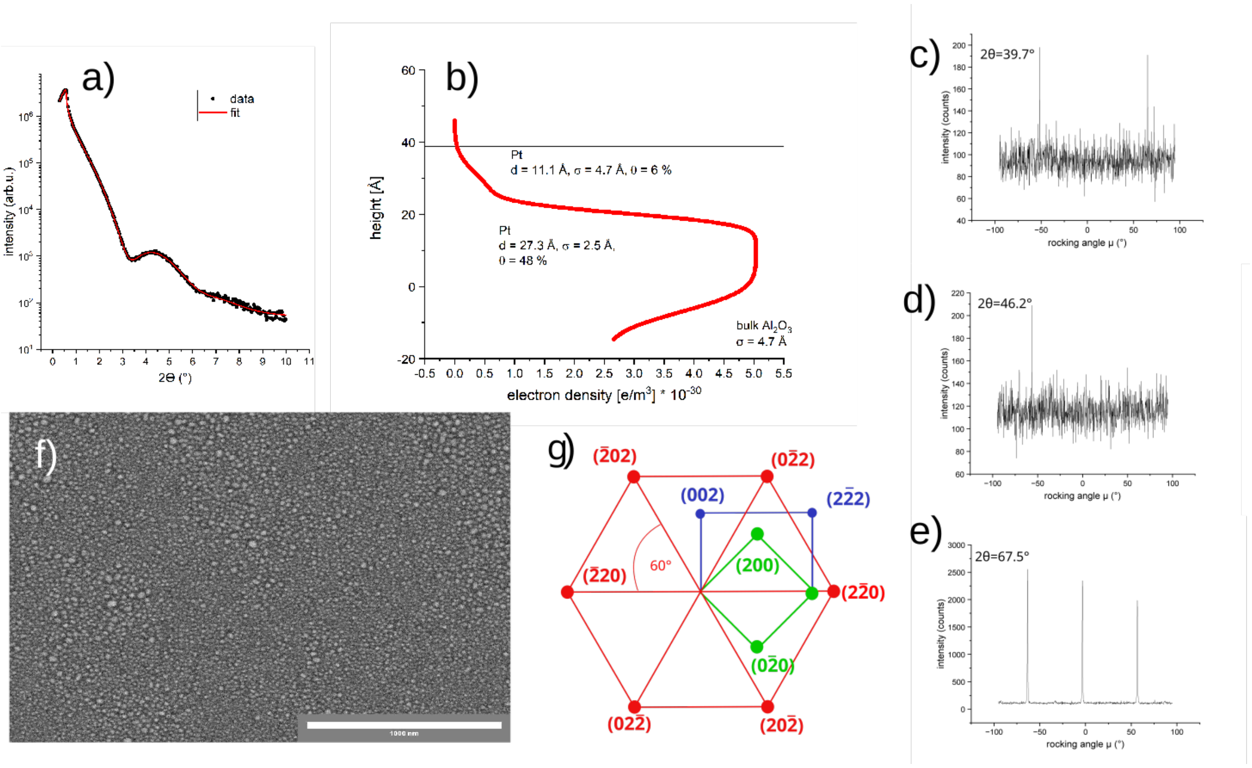

Figure S6: For the Pt sample, a) XRR data with fits, b) electron density from the XRR fit. c) to e) are in-plane XRD scans. The setup is set to detect c) {100} planes, d) {111} planes and e) {110} planes (orthogonal to the surface). The used 2θ angles are given (for Cu K-α radiation). f) is an SEM image of the sample. g) is a combination of the in-plane reciprocal lattice for nanoparticles with (111) orientation (red), (200) orientation (green) and (220) orientation (blue). All orientations share the $(2\bar{2}0)$ reflex. The 60° spacing of the (111) oriented particles' [220] peaks can be seen in e).

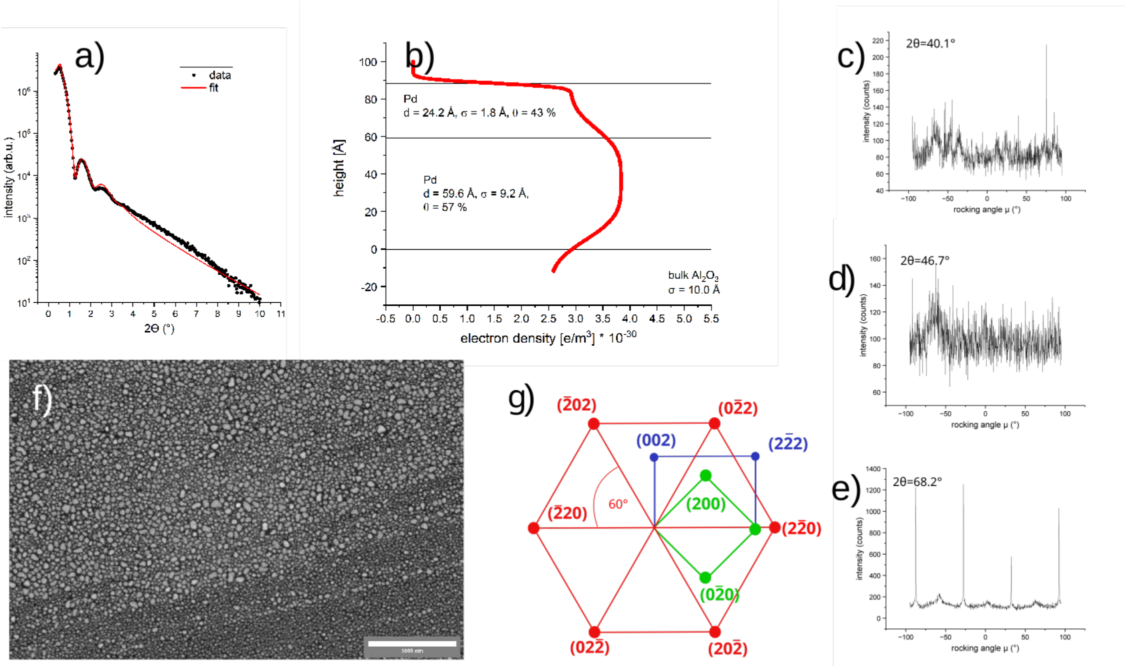

Figure S7: For the Pd NP sample, a) XRR data with fits, b) electron density from the XRR fit. c) to e) are in-plane XRD scans. The setup is set to detect c) {100} planes, d) {111} planes and e) {110} planes (orthogonal to the surface). The used 2θ angles are given (for Cu K-α radiation). f) is an SEM image of the sample. g) is a combination of the in-plane reciprocal lattice for nanoparticles with (111) orientation (red), (200) orientation (green) and (220) orientation (blue). All orientations share the $(2\bar{2}0)$ reflex. The 60° spacing of the (111) oriented particles' [220] peaks can be seen in e).

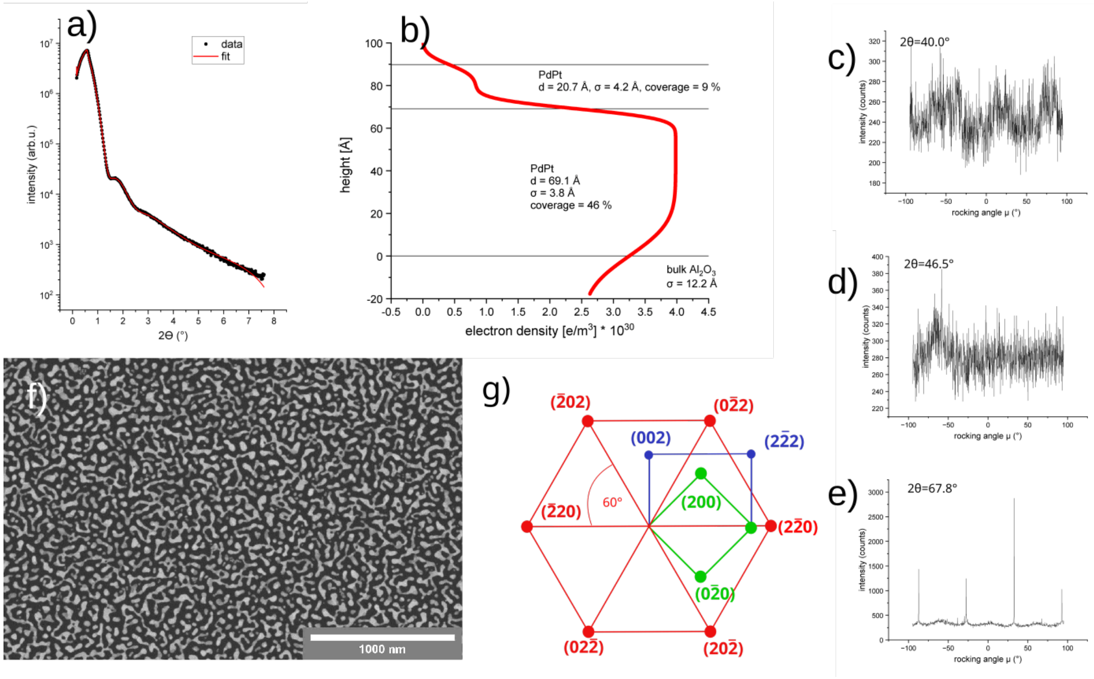

Figure S8: For the PdPt NP sample, a) XRR data with fits, b) electron density from the XRR fit. c) to e) are in-plane XRD scans. The setup is set to detect c) {100} planes, d) {111} planes and e) {110} planes (orthogonal to the surface). Five seconds of integration time were used. The used 2θ angles are given (for Cu K-α radiation). f) is an SEM image of the sample. g) is a combination of the in-plane reciprocal lattice for nanoparticles with (111) orientation (red), (200) orientation (green) and (220) orientation (blue). All orientations share the $(2\bar{2}0)$ reflex. The 60° spacing of the (111) oriented particles' [220] peaks can be seen in e).

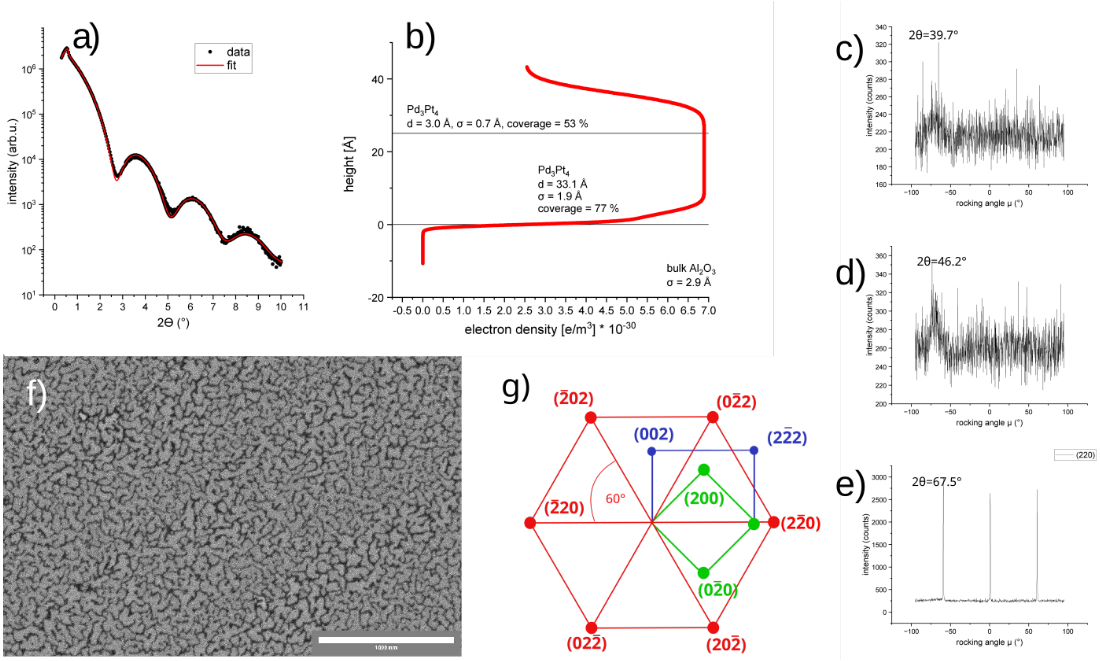

Figure S9: For the Pd_3Pt_4 NP sample, a) XRR data with fits, b) electron density from the XRR fit. c) to e) are in-plane XRD scans. The setup is set to detect c) {100} planes, d) {111} planes and e) {110} planes (orthogonal to the surface). The used 2θ angles are given (for Cu K-α radiation). f) is an SEM image of the sample. g) is a combination of the in-plane reciprocal lattice for nanoparticles with (111) orientation (red), (200) orientation (green) and (220) orientation (blue). All orientations share the $(2\bar{2}0)$ reflex. The 60° spacing of the (111) oriented particles' [220] peaks can be seen in e).

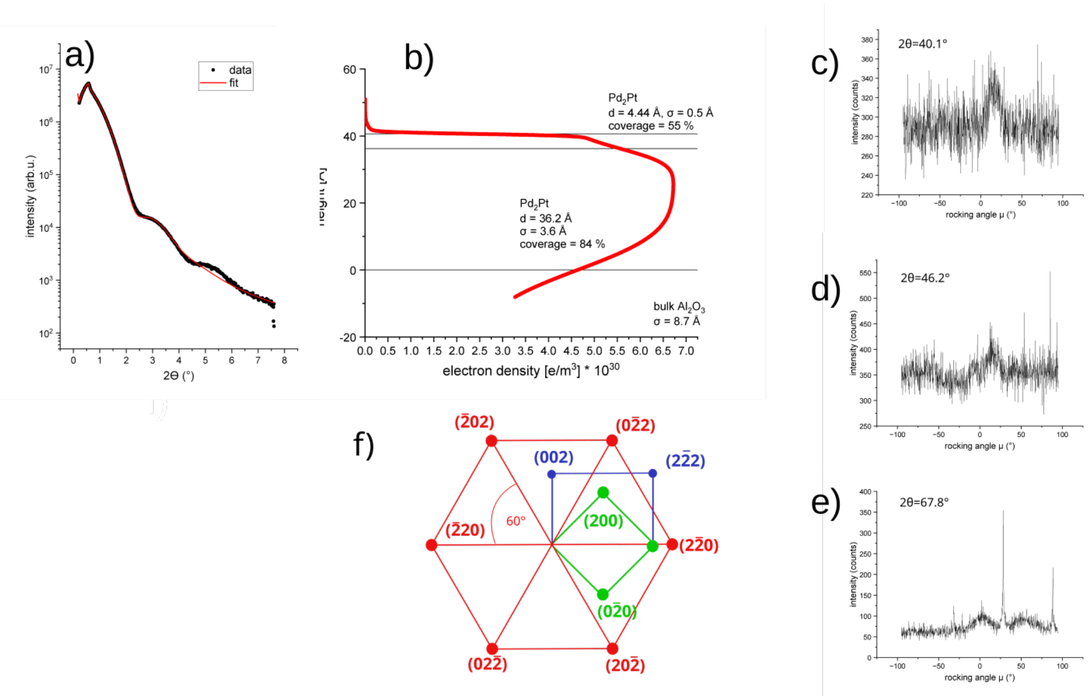

Figure S10: For the Pd_2Pt NP sample, a) XRR data with fits, b) electron density from the XRR fit. c) to e) are in-plane XRD scans. The setup is set to detect c) {100} planes, d) {111} planes and e) {110} planes (orthogonal to the surface). Five seconds of integration time were used. The used 2θ angles are given (for Cu K-α radiation). f) is a combination of the in-plane reciprocal lattice for nanoparticles with (111) orientation (red), (200) orientation (green) and (220) orientation (blue). All orientations share the $(2\bar{2}0)$ reflex. The 60° spacing of the (111) oriented particles' [220] peaks can be seen in e).

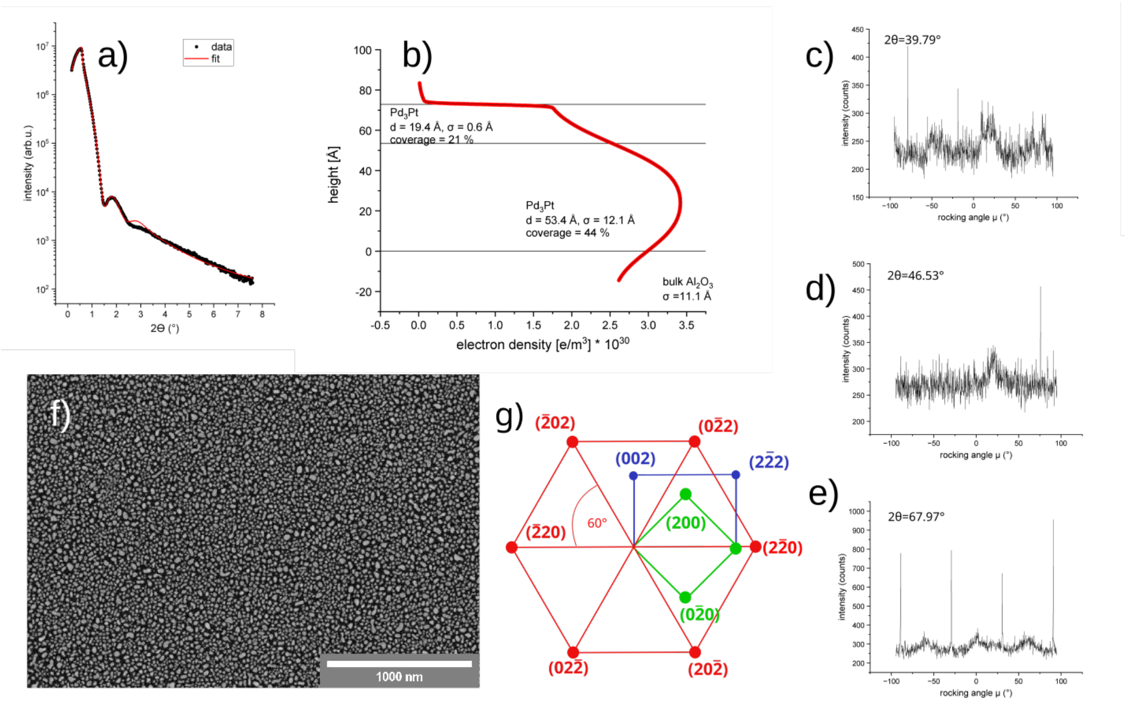

Figure S11: For the Pd_3Pt NP sample, a) XRR data with fits, b) electron density from the XRR fit. c) to e) are in-plane XRD scans. The setup is set to detect c) {100} planes, d) {111} planes and e) {110} planes (orthogonal to the surface). Five seconds of integration time were used. The used 2θ angles are given (for Cu K-α radiation). f) is an SEM image of the sample. g) is a combination of the in-plane reciprocal lattice for nanoparticles with (111) orientation (red), (200) orientation (green) and (220) orientation (blue). All orientations share the $(2\bar{2}0)$ reflex. The 60° spacing of the (111) oriented particles' [220] peaks can be seen in e).

Al_2O_3 Reference: CO Adsorption

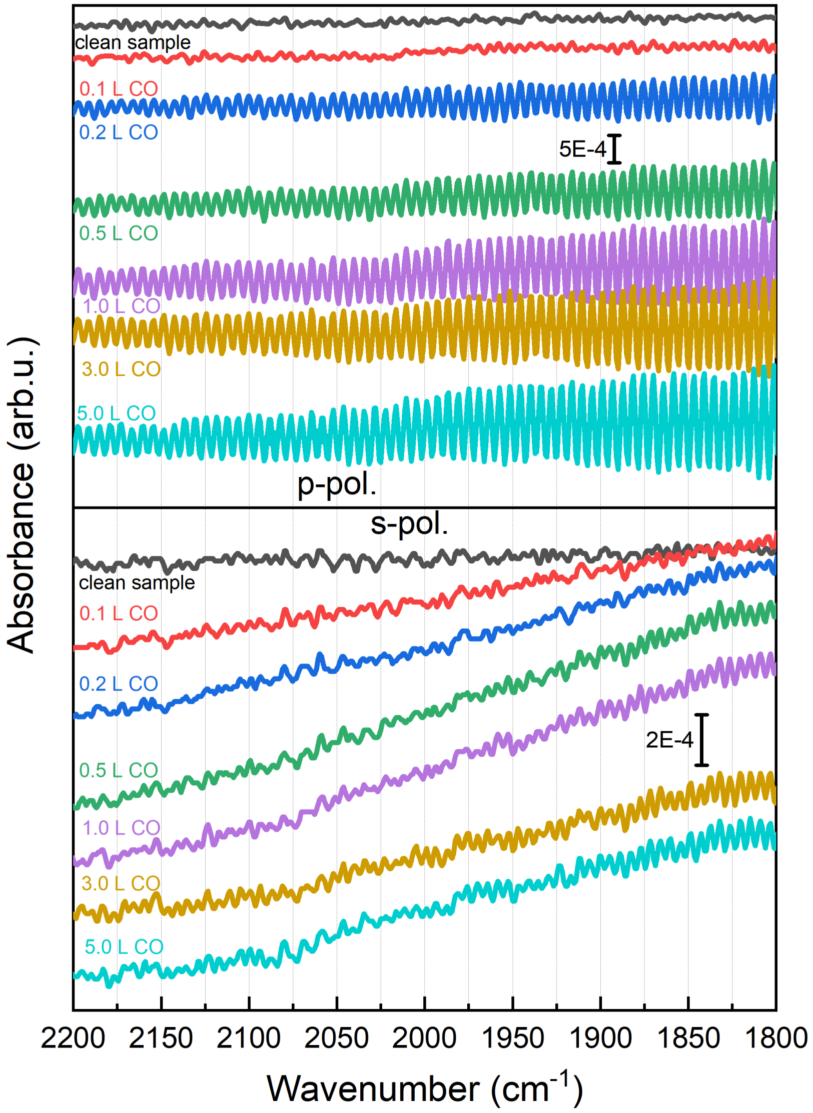

Figure S12: IRRAS data for CO adsorption on a clean Al_2O_3 substrate crystal at 110K. The upper part of the plot contains p-polarized data, the lower part contains s-polarized data. The oscillating noise arose due to mechanical vibrations.

FT-IRRAS Spectra

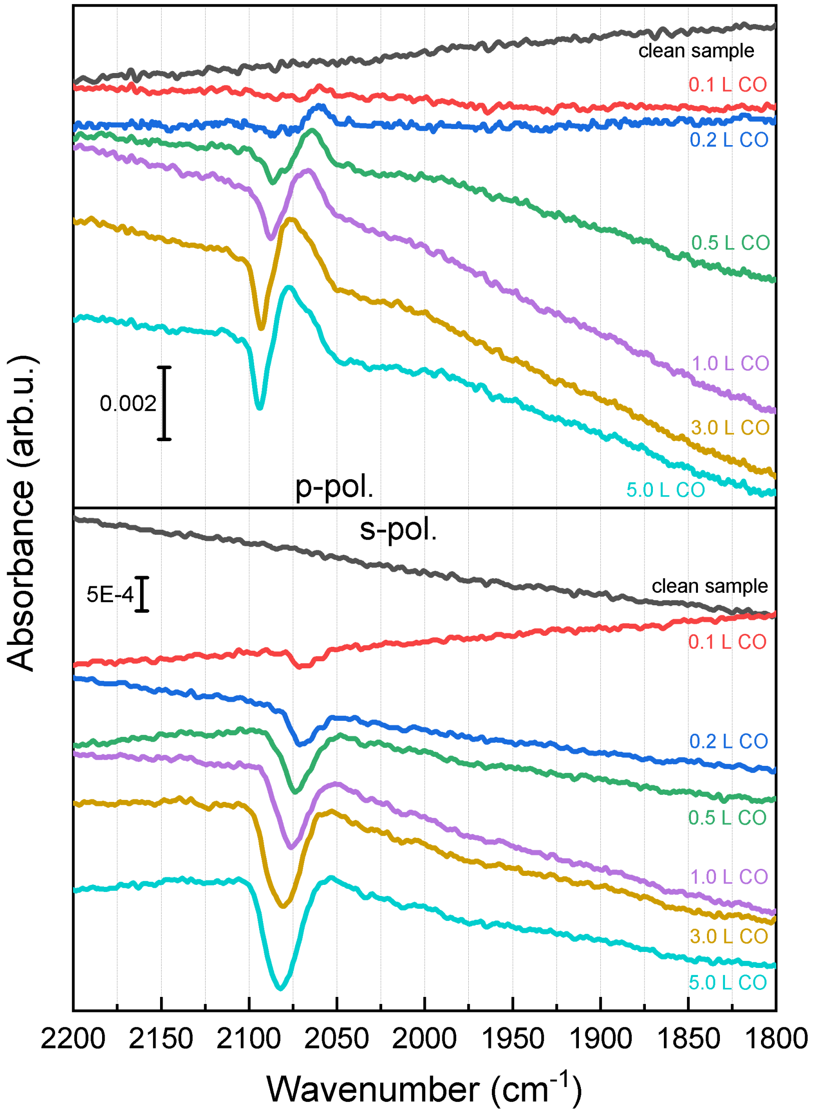

Figure S13: IRRAS data for CO adsorption on Pt nanoparticles on Al_2O_3 at room temperature.

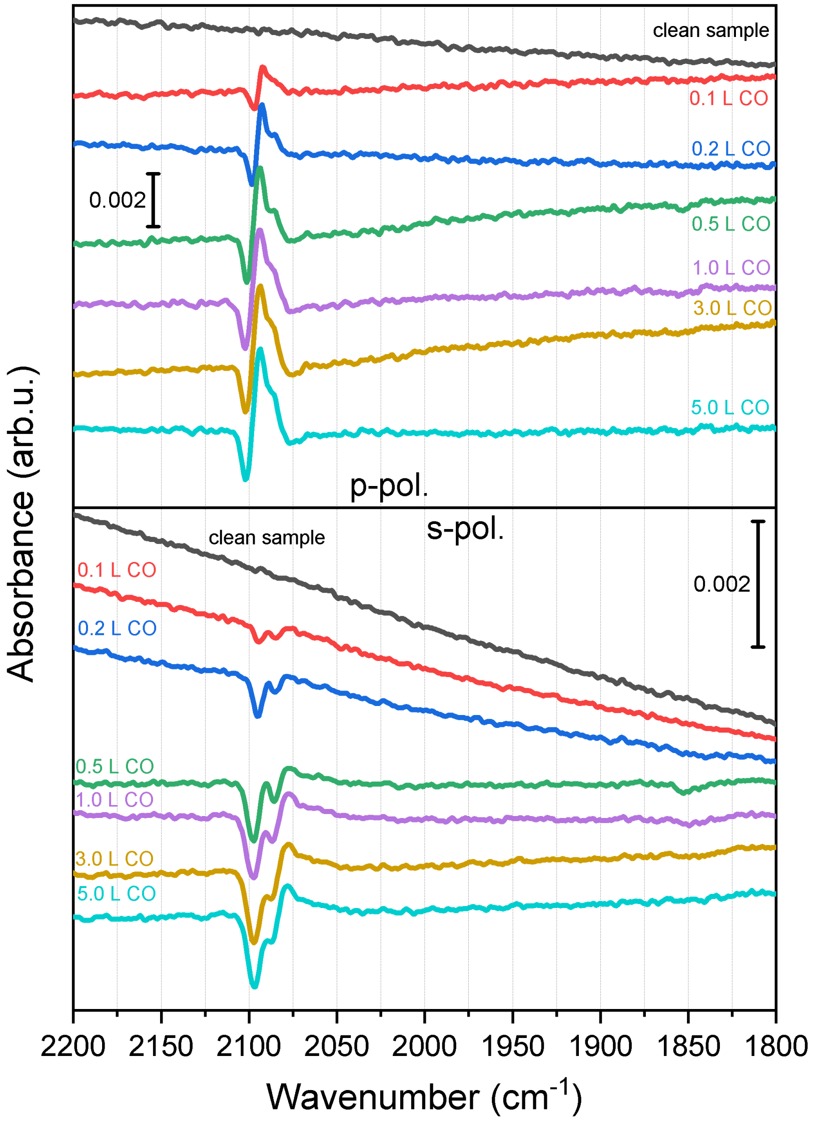

Figure S14: IRRAS data for CO adsorption on Pt nanoparticles on Al_2O_3 at 110K.

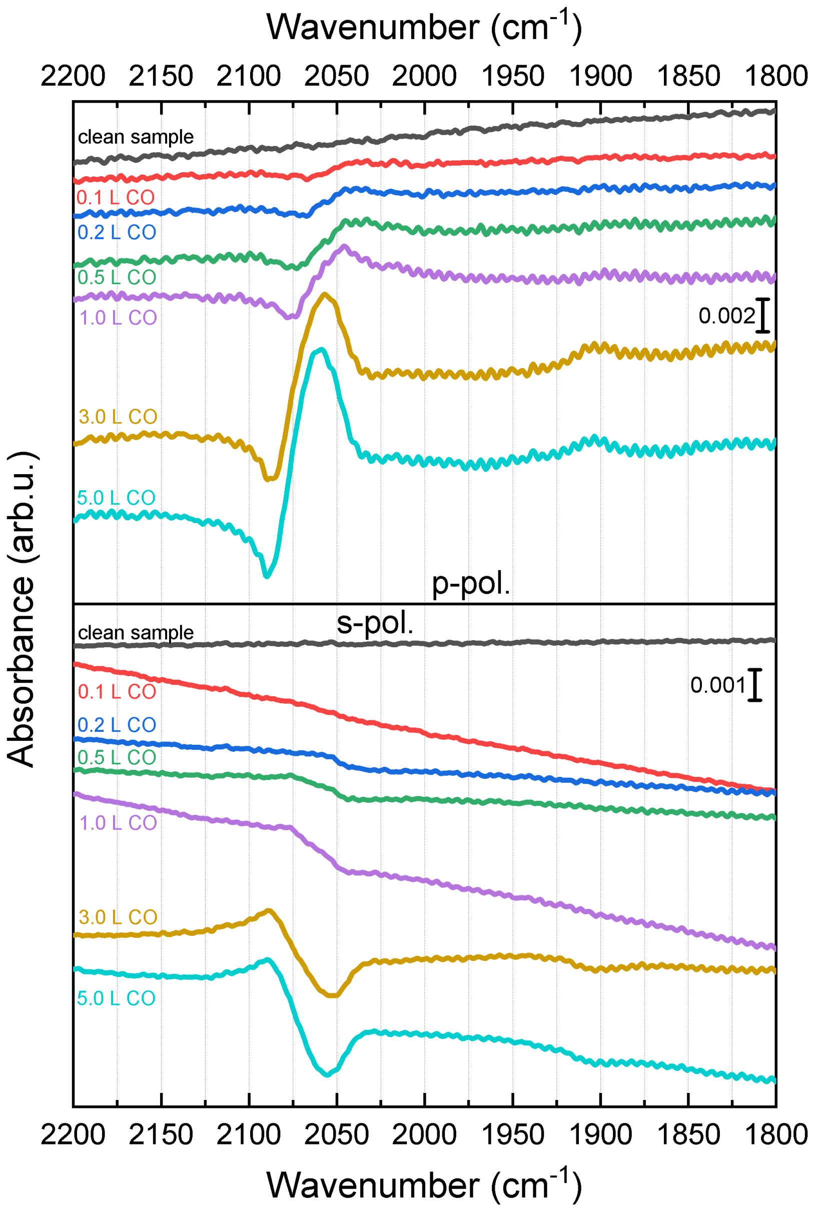

Figure S15: IRRAS data for CO adsorption on Pd_3Pt_4 nanoparticles on Al_2O_3 at room temperature.

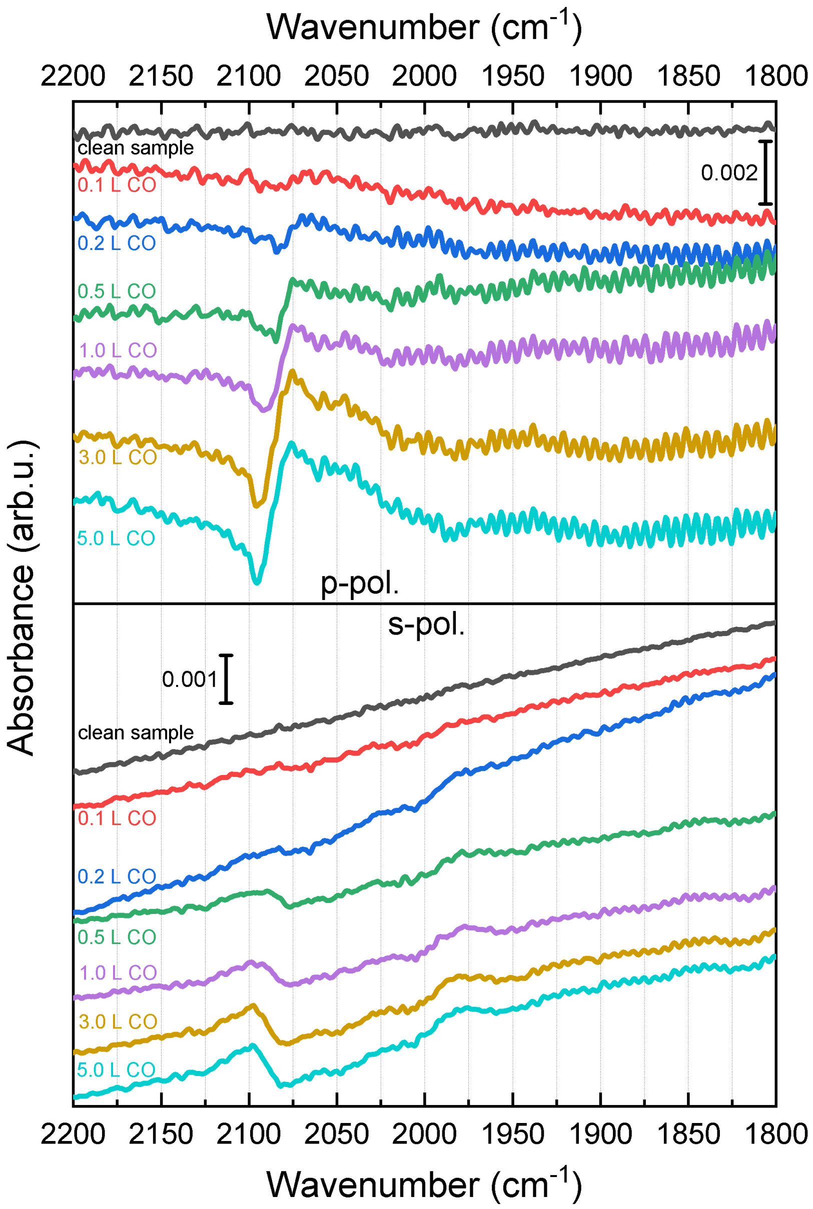

Figure S16: IRRAS data for CO adsorption on Pd_3Pt_4 nanoparticles on Al_2O_3 at 110K.

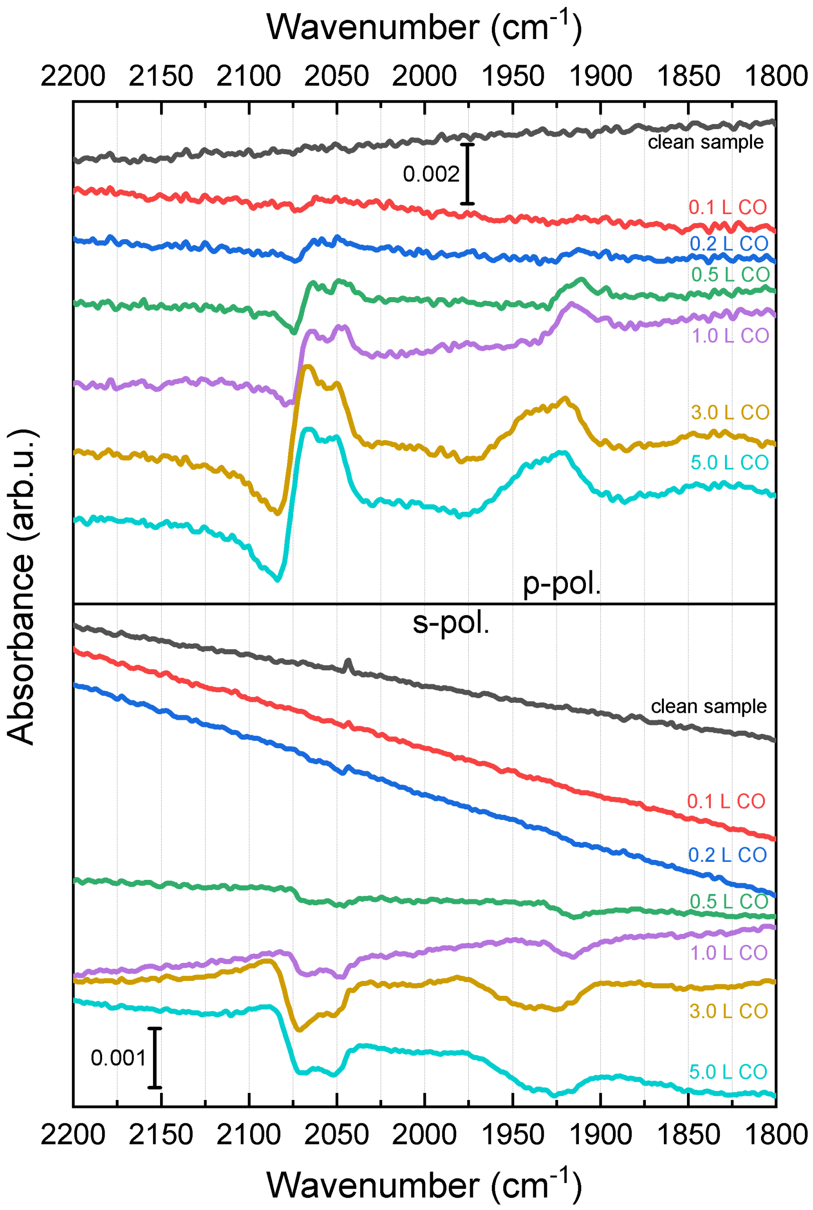

Figure S17: IRRAS data for CO adsorption on PdPt nanoparticles on Al_2O_3 at room temperature.

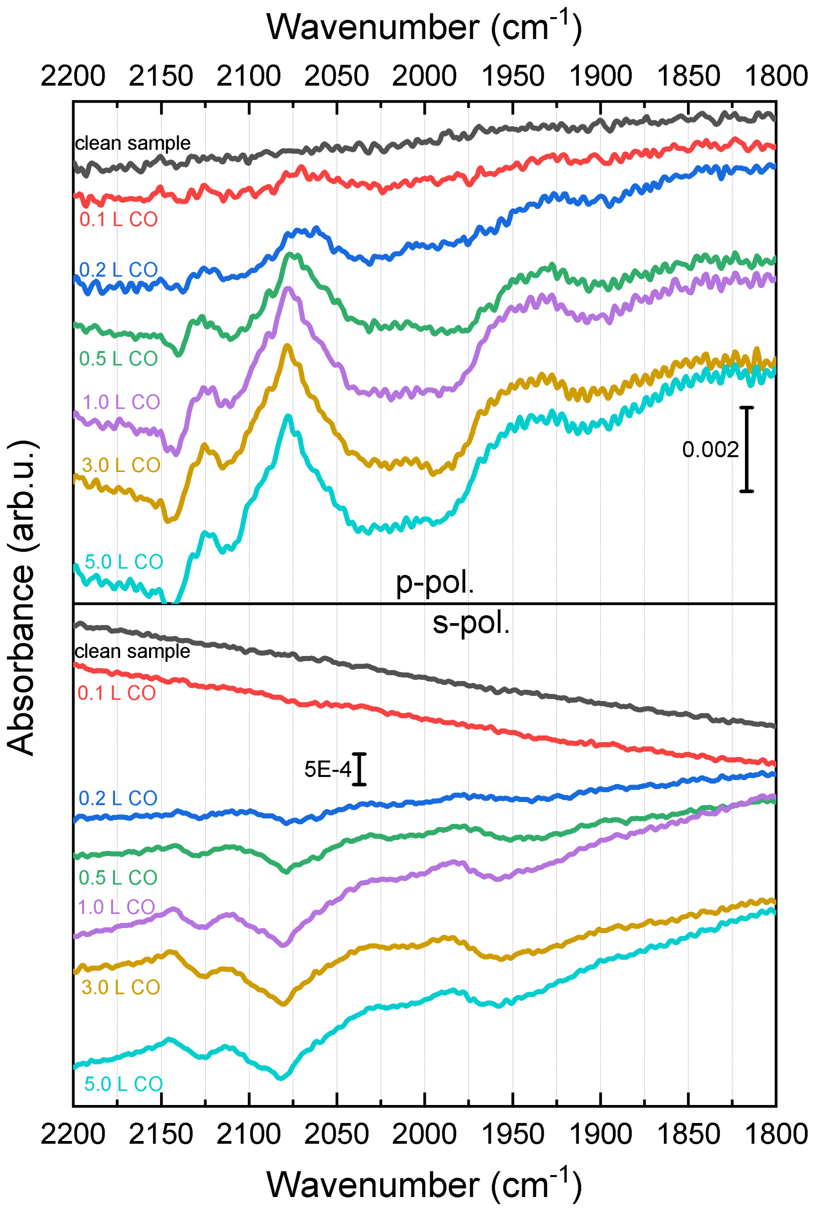

Figure S18: IRRAS data for CO adsorption on PdPt nanoparticles on Al_2O_3 at 110K.

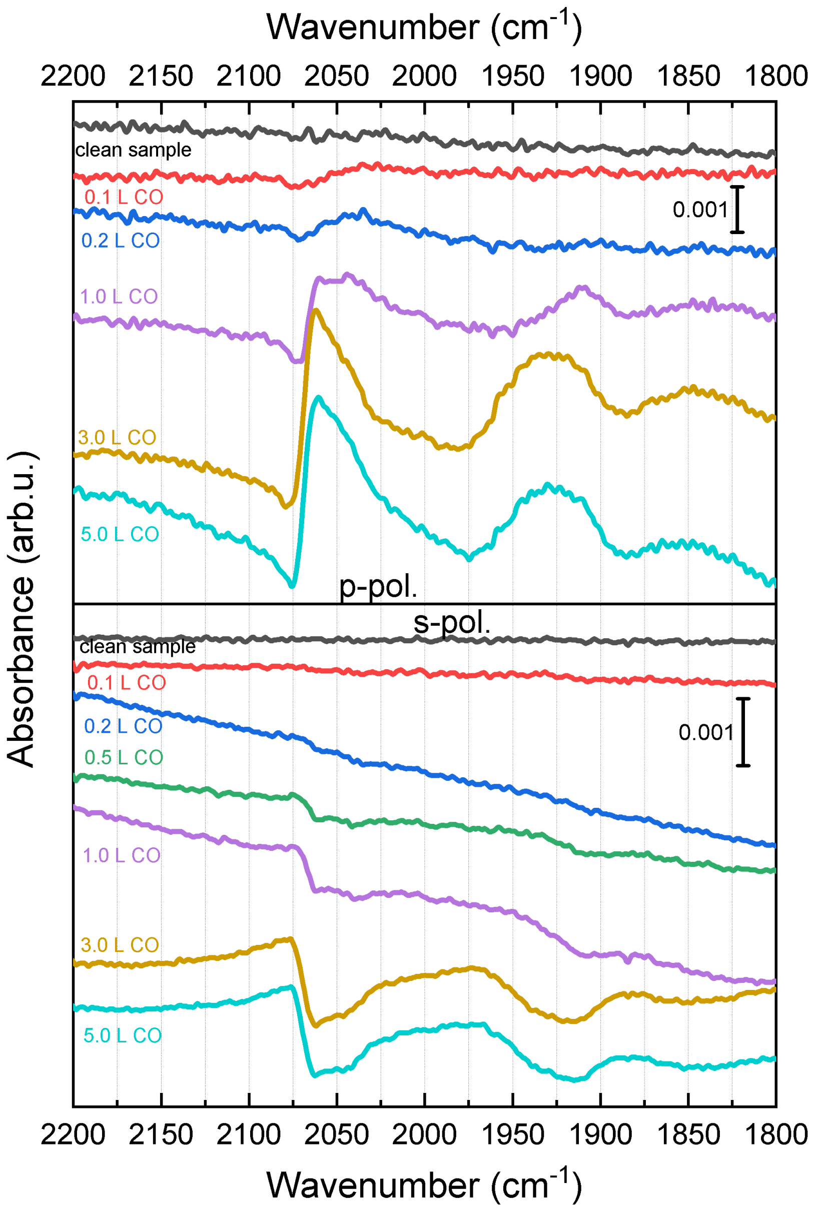

Figure S19: IRRAS data for CO adsorption on Pd_2Pt nanoparticles on Al_2O_3 at room temperature.

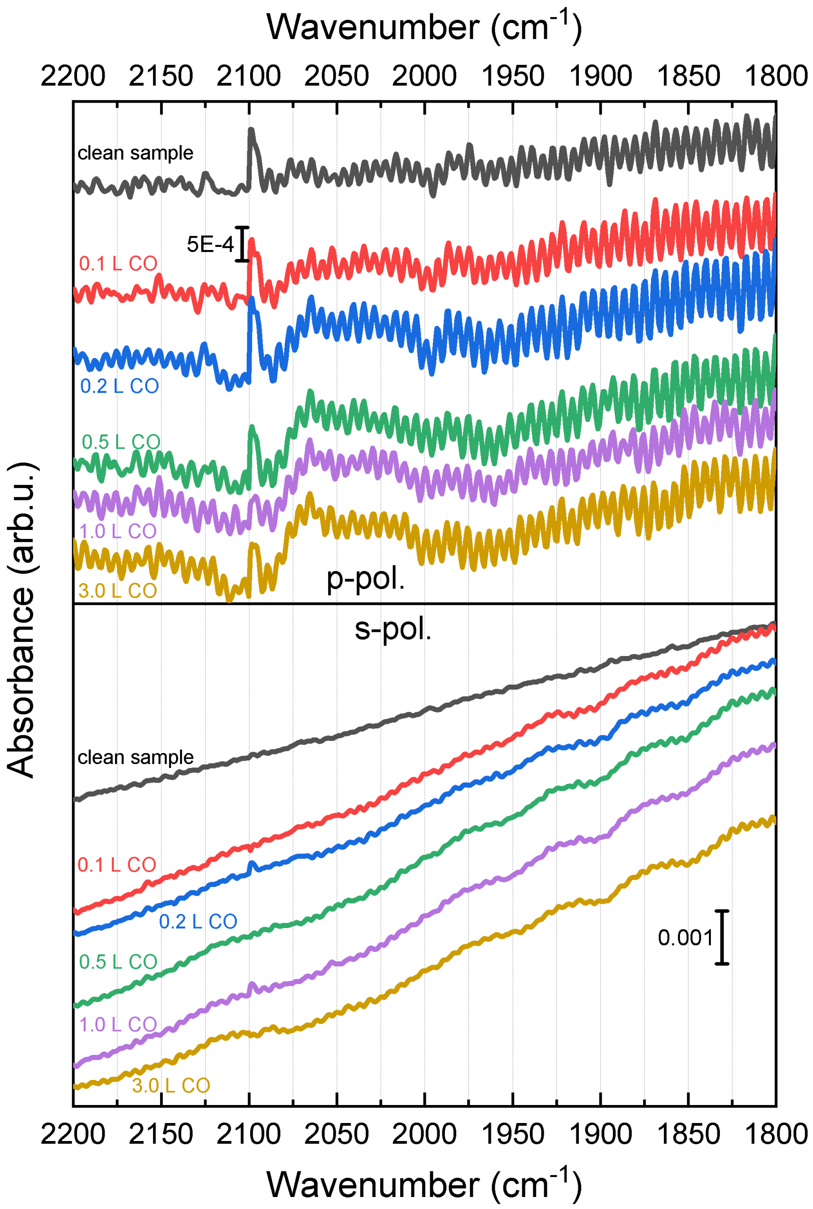

Figure S20: IRRAS data for CO adsorption on Pd_2Pt nanoparticles on Al_2O_3 at 110K.

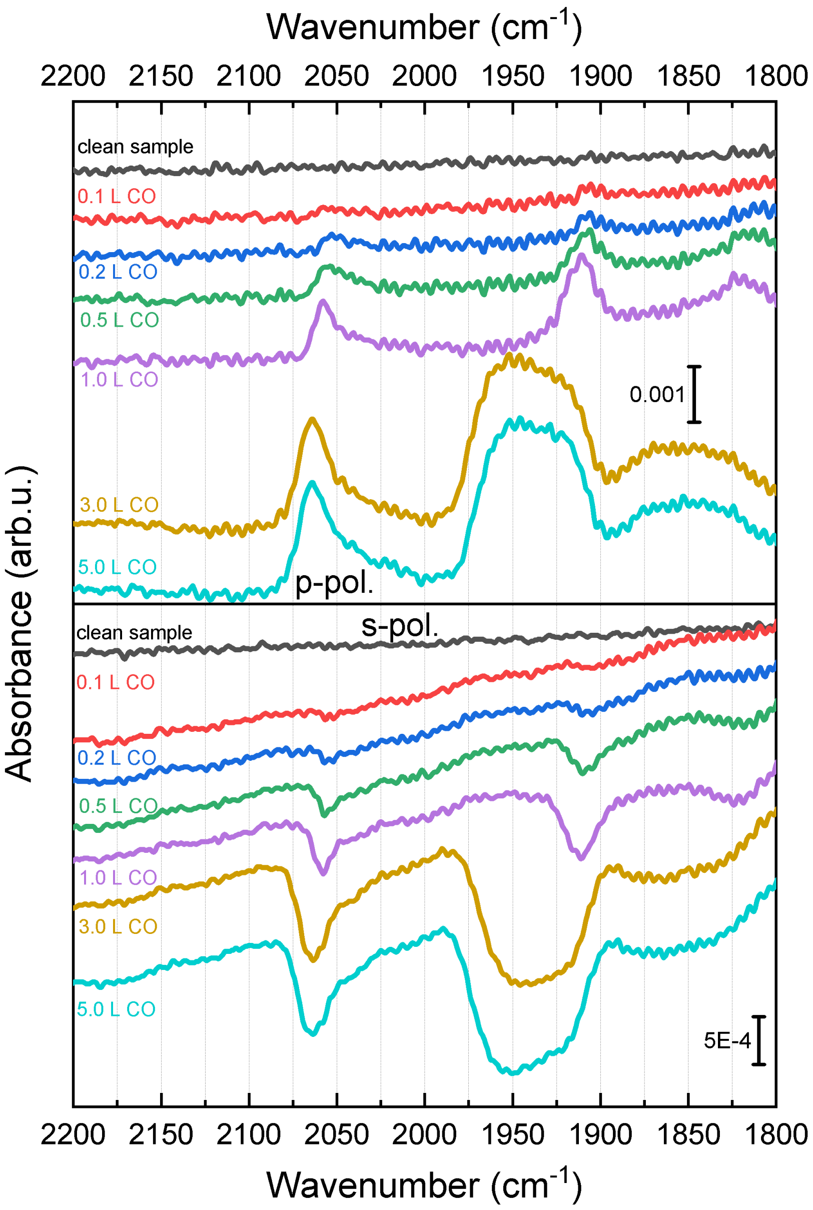

Figure S21: IRRAS data for CO adsorption on Pd_3Pt nanoparticles on Al_2O_3 at room temperature.

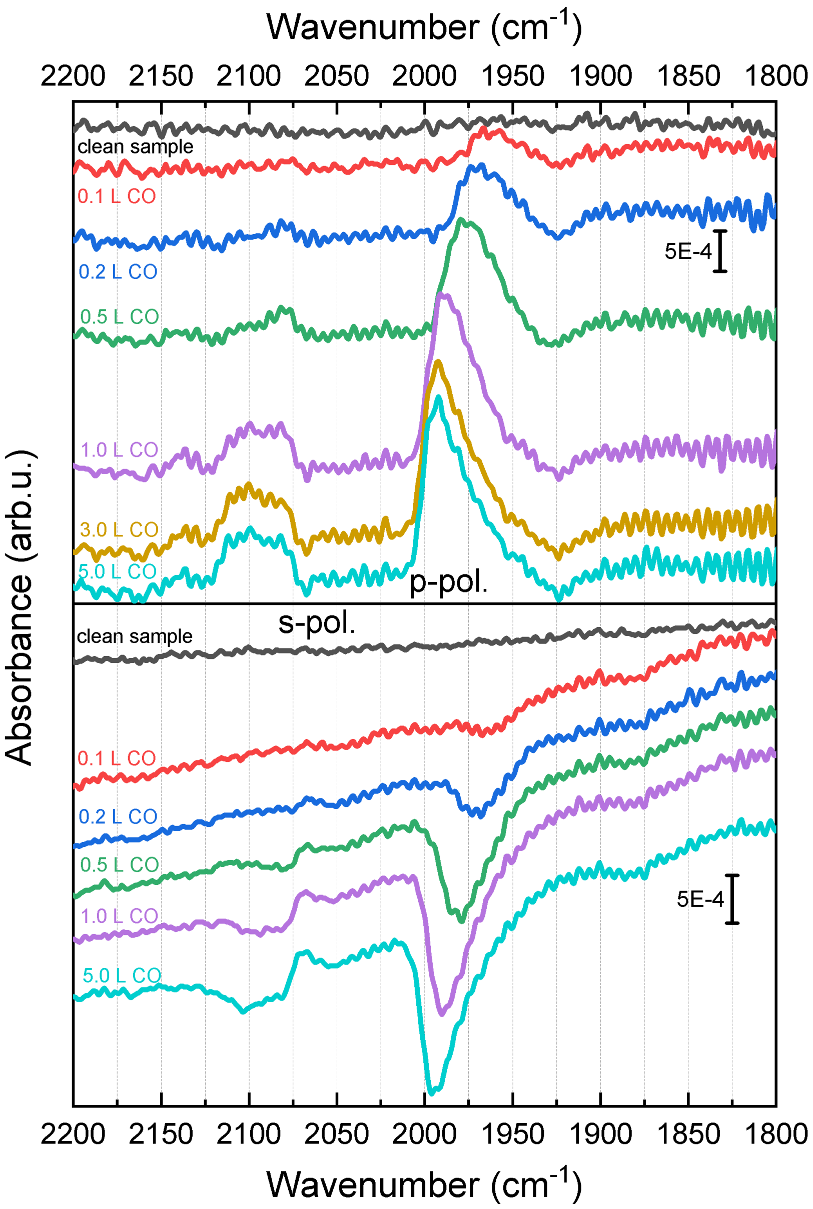

Figure S22: IRRAS data for CO adsorption on Pd_3Pt nanoparticles on Al_2O_3 at 110K.

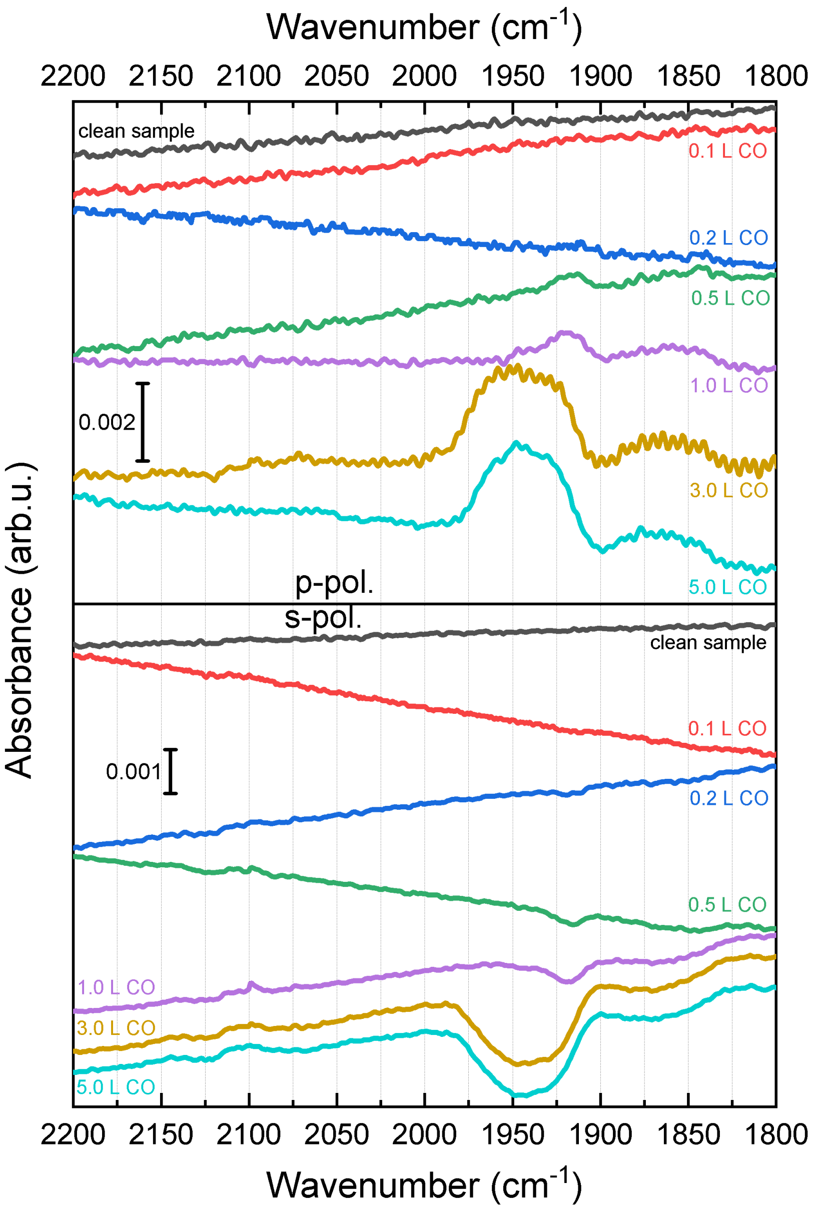

Figure S23: IRRAS data for CO adsorption on Pd nanoparticles on Al_2O_3 at room temperature.

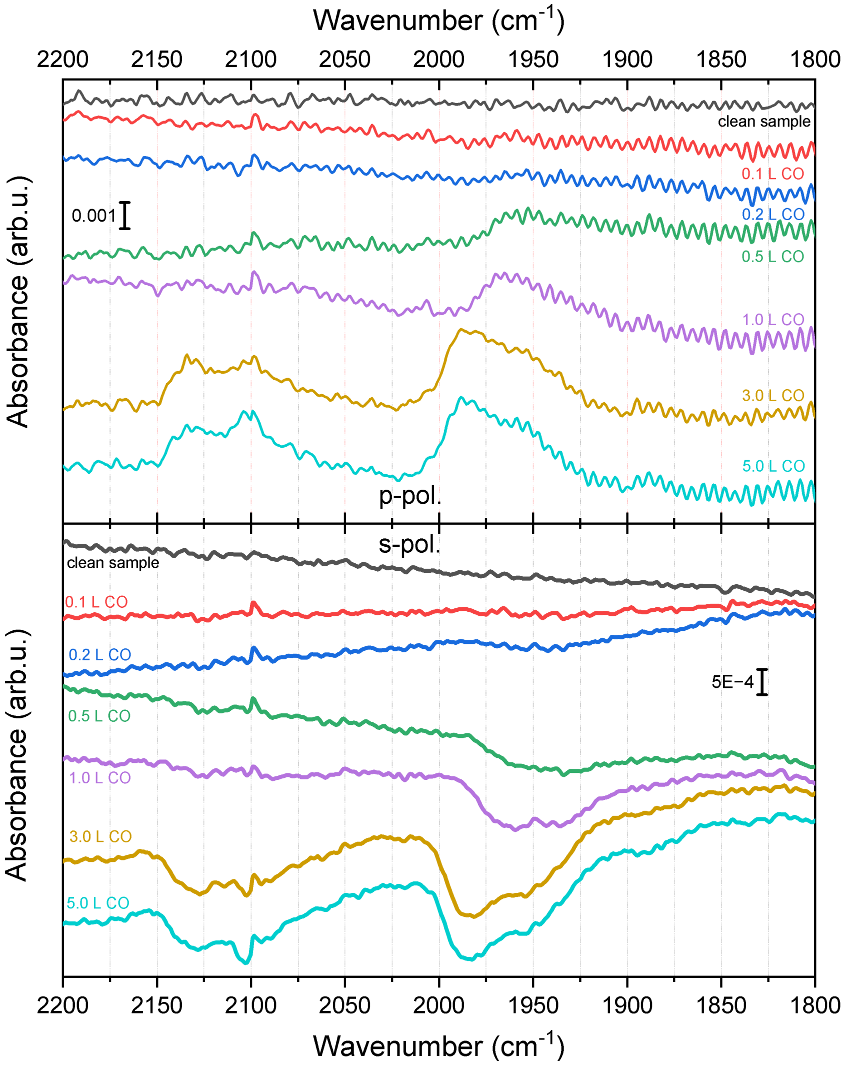

Figure S24: IRRAS data for CO adsorption on Pd nanoparticles on Al_2O_3 at 110K.

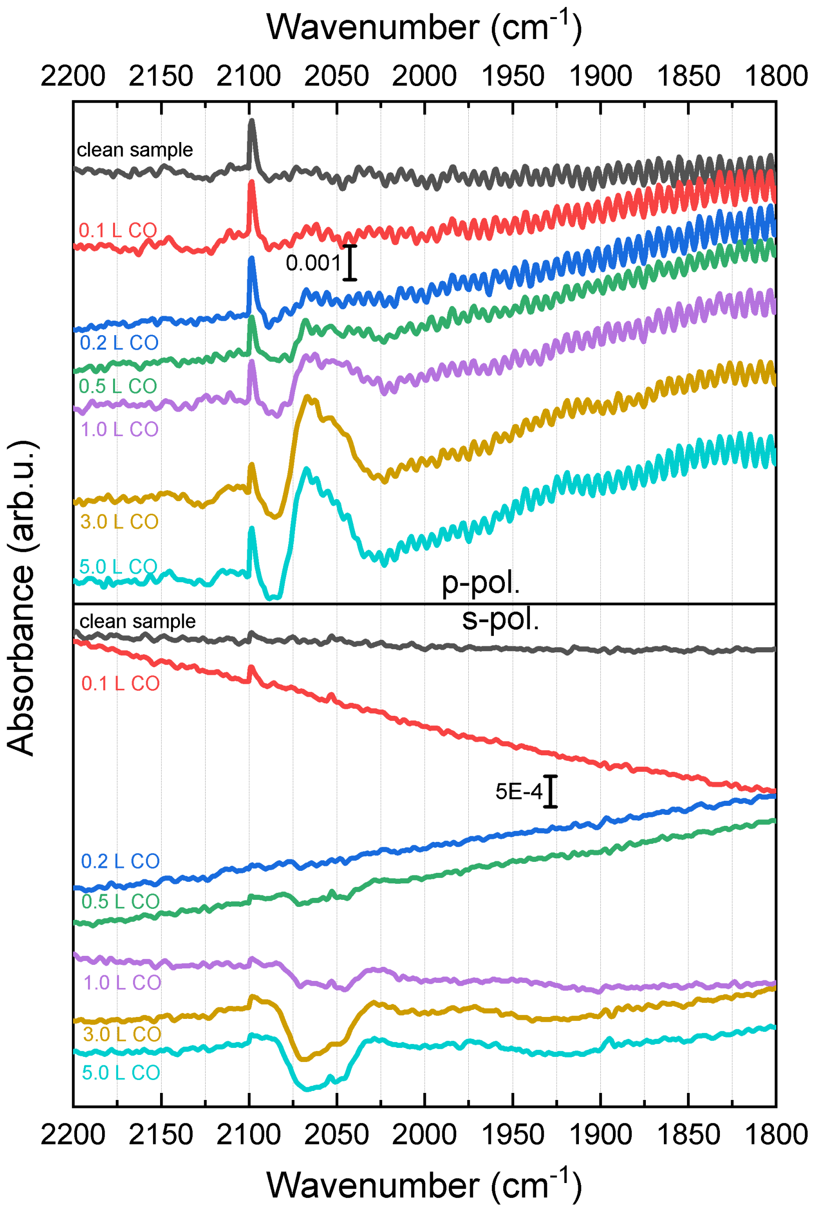

Figure S25: IRRAS data for CO adsorption on PdPt nanoparticles on Al_2O_3 at room temperature after the sample was annealed in hydrogen.

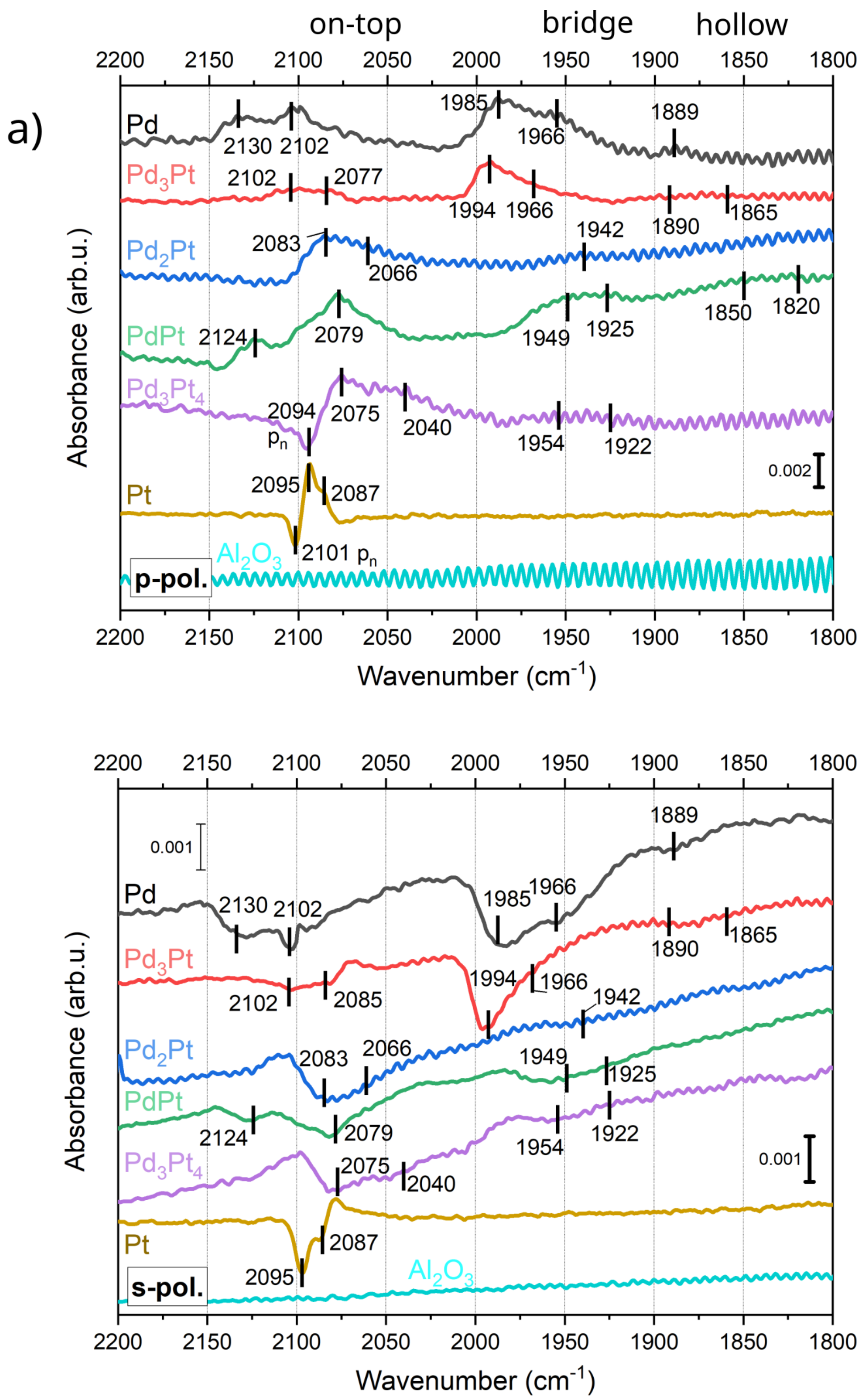

Figure S26: CO adsorption (5 L, dosed at 10−8 mbar partial pressure) at 110 K for the different alloy compositions, with a) p-polarized and b) s-polarized light. In a), all signals that are originating from the p_n part of the light are marked.

References

(1) Mekhemer, G. A.; Zaki, M. I. Low-temperature IR spectroscopy of CO adsorption on calcined supported CeO2: probing adsorbed species and adsorbing sites. *Adsorpt. Sci. Technol.* **1997**, *15*, 377–389.
